# Strain Correlated Linearly Polarized Photoluminescence in WSe_2_/WS_2_ Moiré Superlattices

**DOI:** 10.1021/acs.nanolett.6c02363

**Published:** 2026-09-23

**Authors:** Yuto Urano, Ryo Tamura, Yui Tamogami, Toshikaze Kariyado, Yasumitsu Miyata, Daichi Kozawa, Kenji Watanabe, Takashi Taniguchi, Ryo Kitaura

**Affiliations:** † Research Center for Materials Nanoarchitectonics, 52747 National Institute for Materials Science, 1-1 Namiki, Tsukuba, Ibaraki 305-0044, Japan; ‡ Graduate School of Chemical Sciences and Engineering, Hokkaido University, 5, Kita 8 Nishi, Kita-ku, Sapporo, Hokkaido 060-8628, Japan; § Center for Basic Research on Materials, 52747 National Institute for Materials Science, 1-1 Namiki, Tsukuba, Ibaraki 305-0044, Japan; ∥ Graduate School of Frontier Sciences, The University of Tokyo, 5-1-5 Kashiwanoha, Kashiwa, Chiba 277-8568, Japan; ⊥ Department of Physics, Tokyo Metropolitan University, 1-1 Minamiosawa, Hachioji, Tokyo 192-0397, Japan; # Research Center for Electronic and Optical Materials, 52747 National Institute for Materials Science, 1-1 Namiki, Tsukuba, Ibaraki 305-0044, Japan

**Keywords:** moiré superlattices, transition metal dichalcogenides, excitons, strain, polarization-resolved photoluminescence, valleytronics

## Abstract

Reliable optical control of valley degrees of freedom in moiré excitons requires that the emitted polarization faithfully reflects the underlying valley state. Here, we show that linearly polarized photoluminescence from WSe_2_/WS_2_ moiré excitons is largely insensitive to the excitation linear polarization and therefore does not arise from valley coherence. Automated polarization-resolved photoluminescence and Raman mapping at cryogenic temperature reveal that the degree of linear polarization correlates strongly with local Raman shifts and moiré-exciton observables, identifying strain as the dominant experimental correlate. Linear-regression analysis further shows that strain-related descriptors provide the best prediction of the observed polarization. Guided by theory, we attribute this behavior to strain-amplified breaking of *C*_3_ symmetry in the moiré potential: weak uniaxial strain produces only partial cancellation of locally elliptical emission, yielding a finite far-field degree of linear polarization. These results establish strain as a key control parameter for reliable optical readout in TMD moiré superlattices.

## Graphical abstract

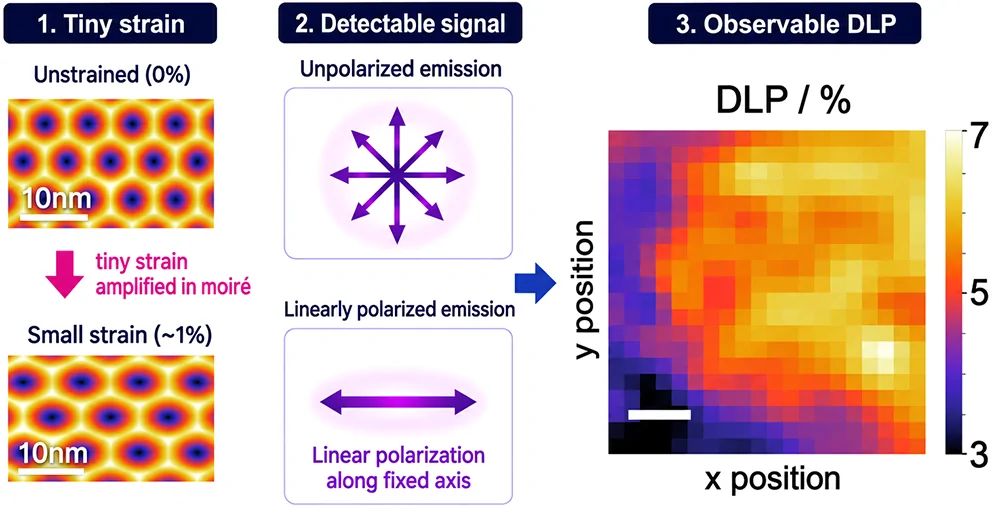

The interplay between valley degrees of freedom (VDF) and light polarization represents a critical aspect of transition metal dichalcogenides (TMDs).^1–4^ Semiconducting TMDs, such as WS_2_ and WSe_2_ in their 2H configuration, exhibit a bandgap at the K and −K points located at the corners of the Brillouin zone.^5,6^ In monolayer TMDs, the breaking of inversion symmetry results in the K and −K points being nonequivalent, and thus the valley index must be specified to describe carriers and excitons in these systems. Because of the optical selection rule arising from 3-fold rotational symmetry, excitons at K and −K selectively couple with right and left circularly polarized light, leading to optical generation and detection of VDF of excitons.^7,8^ The VDF-polarization coupling in TMDs allows us to optically control VDF for next-generation valleytronic devices.^9,10^

The VDF-polarization coupling is also important in moiré superlattices of TMDs. In the moiré superlattices, such as WSe_2_/WS_2_ and twisted MoSe_2_/WSe_2_, interlayer moiré excitons emerge due to the type-II band alignment.^11–13^ Because the electron and hole are spatially separated, interlayer moiré excitons exhibit a long lifetime.^14–16^ Additionally, due to the local structures with 3-fold rotational symmetry (*C*_3_ symmetry) in moiré superlattices, moiré exciton wave packets confined in these high-symmetry locations, A, B, and C, follow *C*_3_ symmetry at K and −K points, leading to the valley-selective optical selection rule coupled with circularly polarized light.^17–20^ The long lifetimes and optical selection rules of moiré excitons enable control of VDF via optical generation and detection.

The potential for optical control relies on a one-to-one correspondence between VDF and light polarization. Circularly polarized light corresponds to the K and −K valleys, while linearly polarized light, a superposition of left and right circularly polarized light, corresponds to a superposition of the K and −K valleys.^21–24^ This one-to-one correspondence enables the preparation of desired valley states and their optical readout. When combined with optical controls such as the valley-selective optical Stark effect, the VDF-polarization correspondence could enable quantum technologies that rely on all-optical generation, manipulation, and detection.

In this work, however, we demonstrate that, unlike monolayer WSe_2_, where the linear polarization in photoluminescence (PL) follows the excitation linear polarization, WSe_2_/WS_2_ moiré exciton shows linearly polarized PL insensitive to the excitation linear polarization, indicating it does not originate from coherent superposition of K and −K valleys. Polarization-resolved PL and Raman maps reveal that the linear polarization correlates with strain across the sample. Guided by a theoretical calculation, we suggest that even a weak strain can distort the moiré potential and weakly break *C*_3_ symmetry, yielding a nonvanishing linear (elliptical) component in the far-field polarization. These findings reveal the strain sensitivity of moiré excitons and highlight an essential consideration for valleytronic device designs utilizing TMD moiré superlattices.

Figure 1a shows an optical image of a triangular WS_2_ crystal, and smaller triangular WSe_2_ crystals grown on the WS_2_ crystal are visible in the inset PL image. In this combination, due to the differences in lattice parameters of each layer, a moiré superlattice can form even when the twist angle between WS_2_ and WSe_2_ is zero. One benefit of direct growth is the clean interfaces between WS_2_ and WSe_2_ with a well-defined interlayer twist angle of 0° or 60°. Polarization-resolved SHG supports assignment of the measured heterobilayer as a near-0° R-type structure: its response is markedly enhanced relative to a reference monolayer WSe_2_ region measured under identical optical conditions, consistent with constructive addition of the layer SH fields; near-60° H-type stacking would instead suppress the heterobilayer signal (Figure S1). Figures 1b and 1c present room-temperature Raman and PL spectra of a WSe_2_/WS_2_ heterostructure grown on an hBN flake. The Raman spectrum measured with 632.8 nm excitation exhibits peaks near 420 and 350 cm^–1^, assigned to the WS_2_ A_1_′ and E′ modes, respectively, and the unresolved WSe_2_ E′ + A_1_′ Raman feature near 250 cm^–1^.^7,25,26^ A room-temperature PL spectrum shows peaks at 2.00 and 1.65 eV, which arise from 1s exciton resonance of WS_2_ and WSe_2_, respectively.^27–30^ A PL spectrum measured at 3.5 K (Figure 1d) shows three low-energy resonances in the interlayer moiré-exciton range.^31,32^ Their collective identification as the interlayer moiré-exciton manifold is well supported; representative microscopic interpretations of the individual resonances are summarized in Figure S2 and its accompanying discussion. All Raman and PL spectroscopy results discussed above are consistent with the formation of a WSe_2_/WS_2_ moiré superlattice.

**Figure 1:**
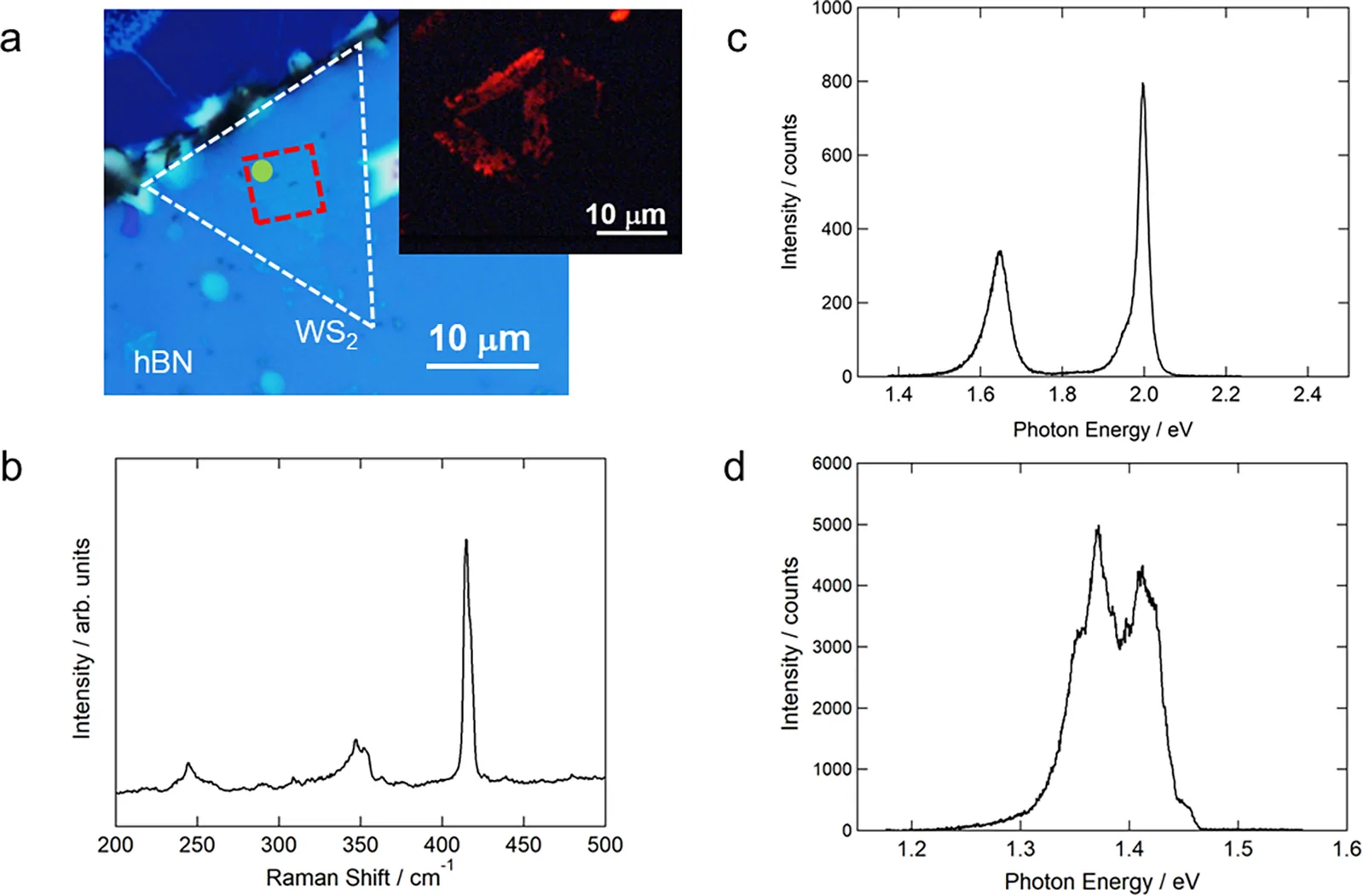
(a) Optical and PL images of the CVD-grown WSe_2_/WS_2_ sample on hBN. The green marker indicates the position at which the spectra in panels (b)–(d) were acquired. The white triangle corresponds to a monolayer WS2 grown on hBN. The dashed square marks the region used for the two-dimensional mapping measurements shown in Figures 2c,d and 3a–f. (b) Raman and (c) PL spectra acquired at room temperature. (d) Moiré-exciton PL spectrum acquired at 3.5 K.

Accurate excitation with polarized light and PL polarization measurements are crucial for controlling VDF in TMD-based heterostructures. To achieve this, we utilized an optical system, as illustrated in Figure 2a. The polarizer and half-wave plate, located directly below the beam splitter, determine the polarization of the excitation light—either vertical or horizontal—before the beam is focused onto the sample. The PL emitted from the sample passes directly to the other half-wave plate and polarizer without any mirrors or optical devices that could disrupt its polarization. Additionally, we incorporated a He–Ne laser for measuring Raman spectra in addition to PL spectra. The control of the sample stage, rotation of half-wave plates, and the on/off operation of the shutters for both the Ti-sapphire and He–Ne lasers are automated in our optical system, enabling fully automated polarization-resolved PL and Raman mapping of samples.

**Figure 2:**
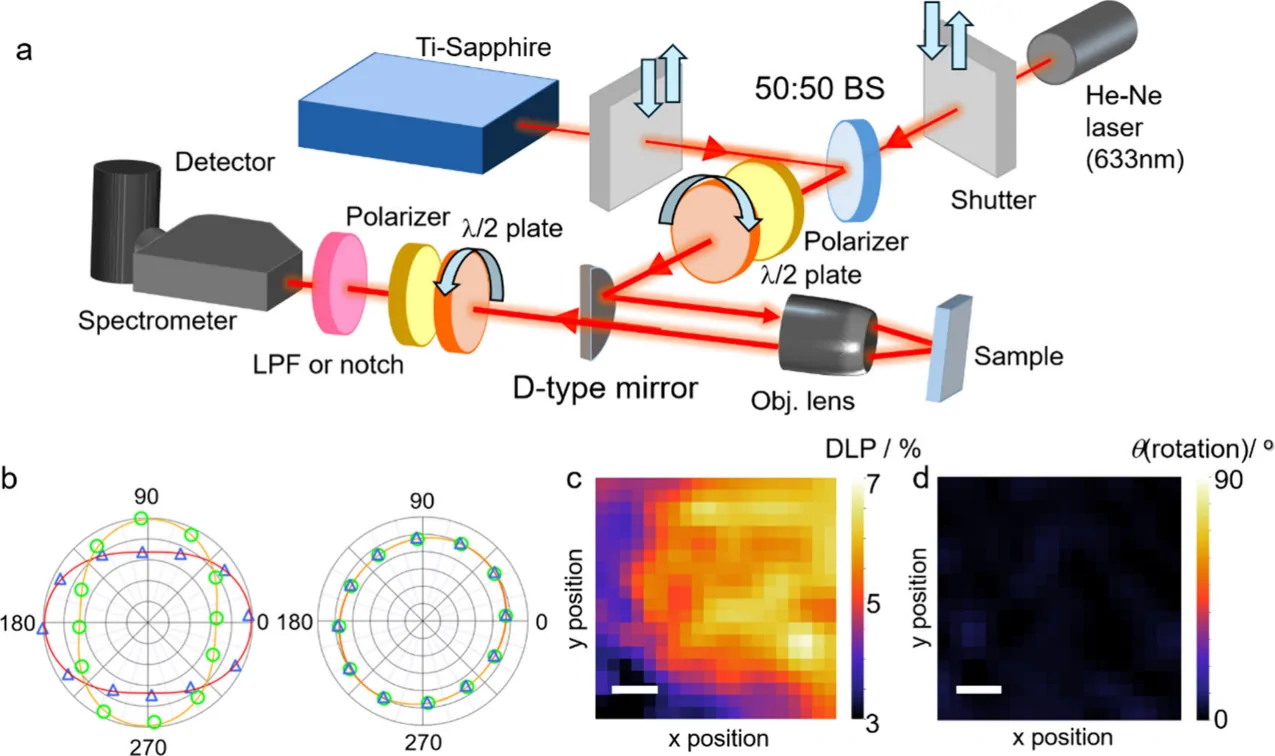
(a) Schematic representation of the optical system used. He–Ne and Ti-sapphire lasers, operating at 633 and 740 nm, respectively, were used for Raman and PL measurements. BS represents a beam splitter. (b) Polar plots measured on monolayer WSe_2_ (left) and the WSe_2_/WS_2_ moiré heterobilayer (right) under the two orthogonal excitation polarizations. The 0° and 90° axes denote the horizontal and vertical polarization directions with respect to the optical bench. (c) and (d) show a 2D map of DLP in % and polarization rotation, θ­(rotation), in degrees upon change of excitation polarization. Mapping was performed over a 5.3 × 5.3 μm region of the sample, as shown in Figure 1a. Scale bars: 1 μm.

Before measuring the WSe_2_/WS_2_ moiré sample, we conducted polarization-resolved PL spectroscopy of monolayer WSe_2_. As shown on the left-hand side of Figure 2b, the PL from WSe_2_ is linearly polarized, exhibiting a degree of linear polarization (DLP) of 18.8%. The DLP is defined as (*I*_0_ – *I*_90_)/(*I*_0_ + *I*_90_), where *I*_0_ and *I*_90_ represent the PL intensities with polarizations parallel and perpendicular to that of the excitation light, respectively. To determine whether the origin of the DLP stems from a superposition of K and −K valleys, we also measured the light polarization of PL using horizontal excitation. As shown in the figure, the polarization direction rotates by 90° when it is switched from vertical to horizontal. The change in polarization direction clearly shows that the emission polarization aligns with the excitation polarization, consistent with the generation and detection of the K and −K superposition states using linearly polarized light. The DLP of 18.8% is also consistent with previously reported values.^33,34^

In contrast, the polarization of PL from the WSe_2_/WS_2_ moiré sample is insensitive to the excitation light polarization. As illustrated on the right-hand side of Figure 2b, the polarization direction remains unchanged regardless of the excitation polarization; it does not align with either the vertical or the horizontal direction. The DLP map shown in Figure 2c demonstrates that a finite DLP of 3% to 7% is present over a broad range of the sample, while the rotation of the polarization direction by altering the excitation polarization is nearly zero in all places (Figure 2d). The polarized PL that is insensitive to excitation polarization differs significantly from those observed in WSe_2_, indicating it likely originates from a different source other than VDF. We do not claim that fixed-axis strain-induced DLP is exclusive to moiré materials, because deliberately strained monolayer WSe_2_ can also exhibit strain-axis-defined linearly polarized emission.^35^ The distinctive feature here is its appearance under weak, unintentional residual distortion, whereas the monolayer WSe_2_ control exhibits excitation-following DLP under the same measurement protocol.

To address the origin of light polarization in PL, we have conducted polarization-resolved PL and Raman mapping of the sample. It is important to note that mapping measurements can take one to 2 days, and drift correction is necessary for obtaining accurate spectral mapping (see Figure S3 for details). In addition to the maps shown in Figures 2c and d, we produced maps for six quantities, including the WS_2_ A_1_′ Raman frequency, the WSe_2_ Raman descriptor, moiré exciton PL intensity (*I*(moiré)), moiré exciton PL peak position (*E*(moiré)), WSe_2_ intralayer trion intensities (*I*(X^–^)), and polarization direction of moiré exciton PL (θ­(axis)). As shown in Figures 3a–f, although the WSe_2_/WS_2_ moiré sample was prepared through direct CVD growth, variations in values across all maps indicate inhomogeneous strain and doping. The Raman shifts contain both carrier-density-related and lattice-distortion-related components. Their maps can therefore share spatial features with *I*(X^–^) through the former component and with DLP through the latter, without requiring a strong direct DLP–*I*(X^–^) relationship. This component-resolved interpretation is supported by the carrier-corrected analysis in Figure S4 and the accompanying discussion. The carrier-corrected WS_2_ A_1_′ residual corresponds to an order-of-magnitude relative strain-equivalent variation of approximately 0.20% (1σ), rather than an absolute strain map (Figure S4).

**Figure 3:**
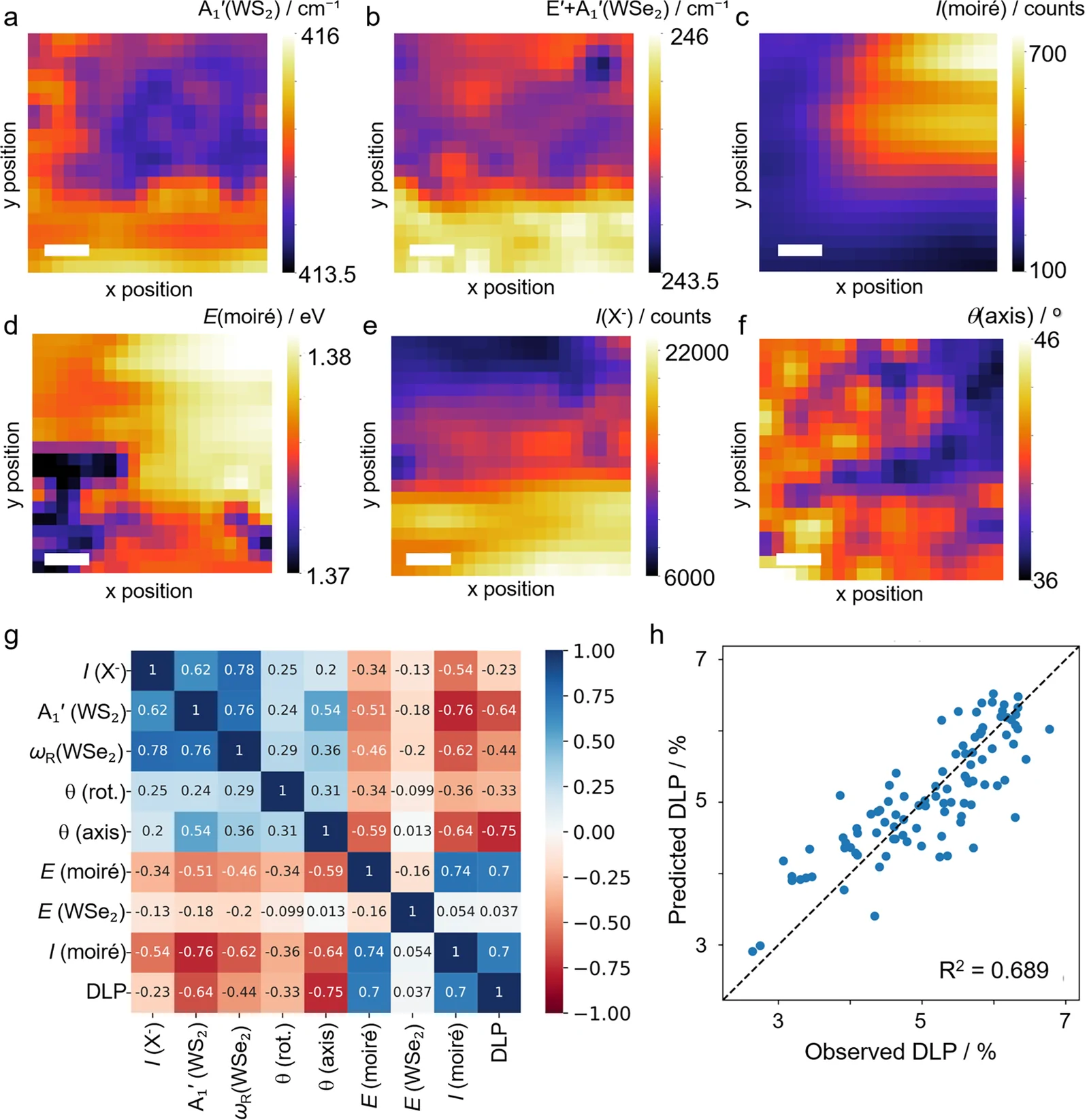
(a)–(f) 2D maps of the WS_2_ A_1_′ Raman frequency, the WSe_2_ Raman descriptor *ω*_R_(WSe_2_) (the peak frequency of the unresolved E′ + A_1_′ feature near 250 cm^–1^), moiré-exciton PL intensity, mean moiré-exciton energy, trion intensity, and moiré-exciton emission-axis angle, respectively. All panels correspond to the same 5.3 × 5.3 μm region, spatial origin, orientation, and analysis grid as Figure 2c,d and were measured at 3.5 K. Scale bars: 1 μm. (g) Pixelwise Pearson correlation matrix; the sign and magnitude are defined in Supporting Note S1. (h) Out-of-fold predicted DLP (%) versus observed DLP (%) for the 10-fold cross-validated linear-regression model. Each point represents one mapped pixel; the dashed line is y = x, and R^2^ = 0.689. Definitions and statistical interpretation are provided in Supporting Note S1.

To quantitatively analyze map similarities, we calculated Pearson correlation coefficients, which measure the linear correlation between two sets of properties. Figure 3g displays pairwise Pearson correlation coefficients among nine quantities, including *I*(X^–^), the WS_2_ A_1_′ Raman frequency, the WSe_2_ Raman descriptor, θ­(rotation), θ­(axis), *E*(moiré), *E*(WSe_2_), *I*(moiré), and DLP. Note that *E*(moiré) is defined by the average positions of three peaks observed in the moiré exciton region. All diagonal elements are equal to one because the linear correlation of identical properties should equal one by the definition of the Pearson correlation coefficient. The WSe_2_ Raman descriptor and the WS_2_ A_1_′ Raman frequency should both be affected by local strain and doping, and the correlation coefficient of 0.76 between them is reasonably high. Another observation is the positive correlation between the *I*(X^–^) and Raman shifts, as evidenced by the similarity between the *I*(X^–^) and Raman shift maps. The *I*(X^–^) represents the carrier density in the sample, and the positive correlation coefficients indicate that carrier-density variations contribute to both Raman descriptors.^36,37^ Definitions and interpretation of the statistical metrics are provided in Supporting Note S1.

The DLP exhibits correlation coefficients of −0.64 and −0.44 with the Raman descriptors, reflecting the similarities between the DLP map and the Raman-descriptor maps. The correlation with Raman descriptors suggests that the observed DLP is associated with strain in the sample. Additionally, the correlation coefficient with *E*(moiré) is also high, 0.70. This aligns with the strain-induced DLP, as the strain is also expected to influence the moiré exciton peak positions through strain-induced moiré potential modulation (a related discussion is provided below). Also, as discussed later, θ­(axis) is directly linked to the deformation of the strain-induced moiré potential, resulting in a high correlation of −0.75. In contrast, the correlation coefficients with *I*(X^–^) and the peak positions of the intralayer exciton are pretty low. The low coefficient for *I*(X^–^) indicates that the dependence of the DLP does not arise from local Fermi level fluctuations in the WSe_2_/WS_2_ sample. The fluctuation of the Fermi level can locally modulate the carrier density, altering the dynamics of excitons, including trion formation and Auger recombination. The observed low correlation indicates that the PL polarization is not directly related to these dynamics.

Based on the experimental results, we have constructed linear regression models for DLP prediction. To perform feature selection, we calculated prediction accuracy for all combinations of properties using 10-fold cross-validation^38^ (we investigated 2^8^ – 1 = 255 combinations; see Figure S5). The highest prediction accuracy was obtained when using the four quantities: *I*(X^–^), A_1_′ (WS_2_), θ­(axis), and *E*(moiré). These four quantities are also used in all top-10 prediction models, as illustrated in Figure S5, underscoring their importance for understanding the observed DLP. Figure 3h represents the relationship between the actual and predicted values for the test data, along with the R^2^ score. Although the prediction accuracy is not remarkably high, DLP can be predicted using four quantities. Although A_1_′ (WS_2_), θ­(axis), and *E*(moiré) should be closely correlated with strain, *I*(X^–^), which has a small correlation coefficient, is still included in the prediction model. The weak direct correlation between *I*(X^–^) and DLP indicates that the observed DLP is unlikely to be governed primarily by local carrier-density variations.

The WSe_2_/WS_2_ sample used was prepared by direct growth onto an hBN flake. AFM of the hBN-capped directly grown sample shows a comparatively flat upper surface on the optical-spot scale (Figure S6), while bubble- or roughness-induced strain at the buried heterobilayer interface cannot be excluded. Bubble-like contrasts are also evident in the image, likely due to impurities trapped between the top hBN and the WSe_2_/WS_2_ heterostructure; we placed an hBN overlayer after growth to protect the heterostructure from adsorbed impurities. The impurities, along with the subnanometer-scale roughness in the bottom hBN layer—likely resulting from the heating and cooling processes during chemical vapor deposition (CVD) and the differences in thermal expansion coefficients between hBN and SiO_2_—probably contribute to the strain distribution observed in the Raman shift patterns displayed in Figures 3a and 3b.

In moiré superlattices, slight strain can cause significant distortion of the moiré period, d_moiré_. The d_moiré_ is represented by d_WS2_d_WSe2_/(d_WSe2_-d_WS2_), where d_WSe2_ and d_WS2_ correspond to the cell parameters of WSe_2_ and WS_2_, respectively. The d_WSe2_ of 0.328 and the d_WS2_ of 0.315 nm yield^39,40^ a d_moiré_ of 7.95 nm, which matches the reported experimental values well. The derivative of d_moiré_ with respect to d_WS2_ is proportional to (d_WSe2_/(d_WSe2_-d_WS2_))^2^, and the denominator, (d_WSe2_-d_WS2_), is small due to the slight difference between d_WSe2_ and d_WS2_, indicating that a tiny change in d_WS2_ can cause a significant alteration in d_moiré_. For instance, a tensile strain of 0.1% in WS_2_ results in a 2.6% change in d_moiré_ along the direction of the strain, making it 26 times as large as the original strain. When uniaxial strain is introduced, d_moiré_ can be distorted. Figure 4a displays structural models of WSe_2_/WS_2_ under three conditions: without strain and with 1% or 2% tensile uniaxial strain applied to the WS_2_ layer along the armchair direction; the Poisson ratio of WS_2_ is used to construct the structural model.^41^ As illustrated, a tensile strain of 2% in the WS_2_ layer distorts the moiré potential, transforming its shape from circular to ellipsoidal, with an aspect ratio of 2.29. Figure 4b shows the relationship between uniaxial strain and aspect ratio, highlighting that even minor strains can cause a noticeable distortion of the moiré potential.

**Figure 4:**
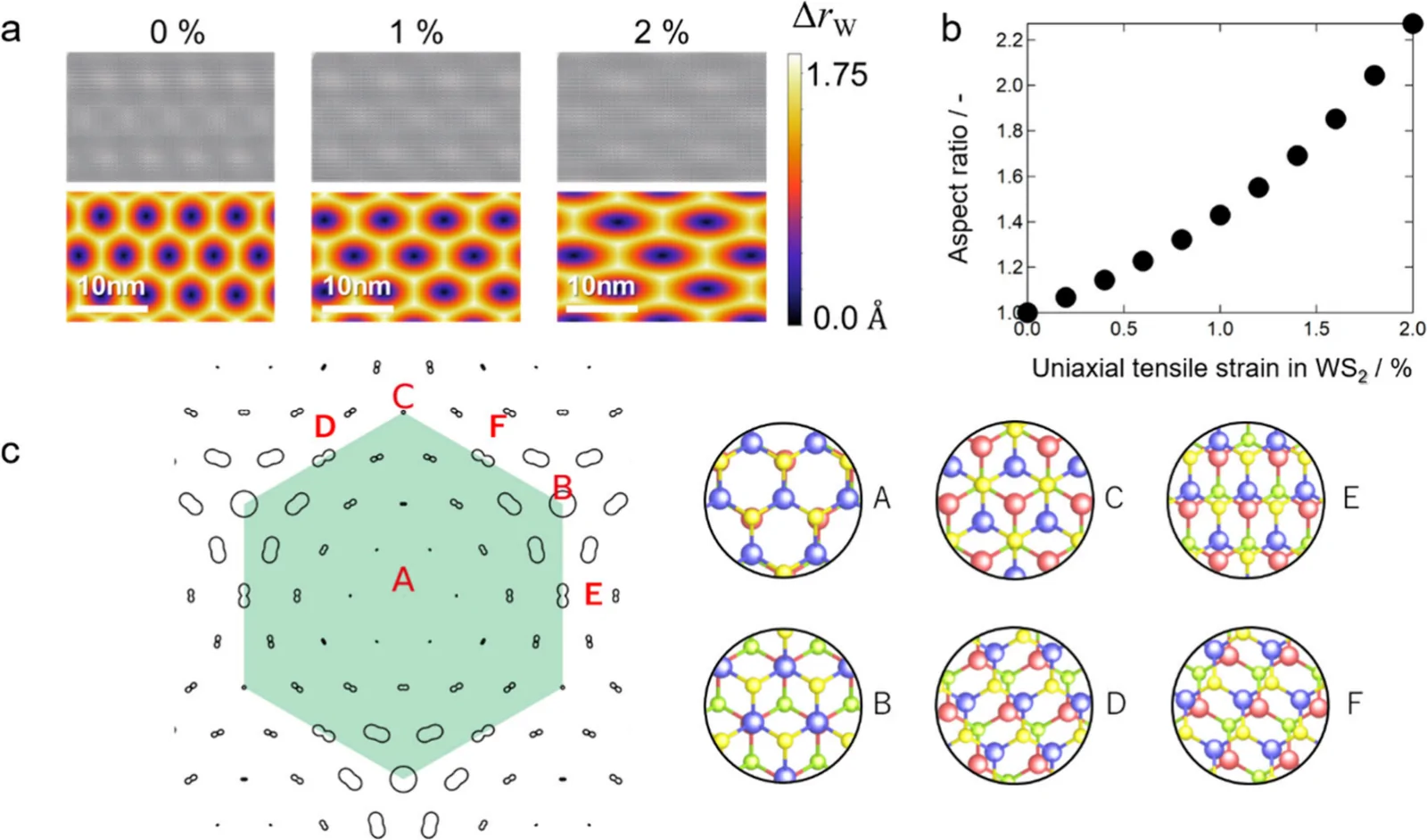
Strain amplification and local optical anisotropy in a near-0° R-type WSe_2_/WS_2_ moiré superlattice. (a) Structural models for 0–2% uniaxial tensile strain applied to WS_2_ (top) and maps of the minimum in-plane W–W registry displacement, Δr_W_ (bottom); a W atom in one layer is paired with the closest W atom in the other layer based on their x–y coordinates, and the lateral distance between them is Δr_W_ expressed in angstroms. The color bar corresponds to Δr_W_. The 2% model is used only to visualize the deformation and is not an estimate of the experimental strain. (b) Aspect ratio of the moiré site versus physical WS_2_ strain, calculated from the lattice mismatch along the two principal directions. The curve in panel (b) is not a fit to the measured DLP. (c) Local transition-matrix anisotropy for the near-0° R-type structure. The green hexagon marks the moiré Wigner–Seitz cell, with the local structures at A–F shown on the right. Blue and red spheres denote W in the top and bottom layers, and yellow and green denote Se and S, respectively. The polar plots show local linear-polarization selectivity. In the *C*_3_-symmetric limit, the three *C*_3_-related contributions cancel in the far field; strain-amplified deformation makes their weights inequivalent, leaving a finite DLP. This local analysis does not evaluate the circular valley selection rule.

The wave functions of the conduction and valence bands can be expressed as exp(ik⃗·r⃗) χ­(r)*u*(r), where exp(ik⃗·r⃗) represents the Bloch phase factor and χ­(r) represents the envelope function, which represents the confinement arising from the moiré potential, and *u*(r) denotes the Bloch function, mainly composed of d-orbitals of tungsten atoms. While the Bloch phase term just gives momentum conservation in optical transitions, χ­(r) and *u*(r) can cause polarization selectivity. As previously discussed, even a small strain can significantly distort the moiré potential, causing the shape of χ­(r) to change from symmetric to ellipsoidal. While χ­(r) does not directly give polarization selectivity in the interband transitions, *u*(*r*) can contribute polarization selectivity through the transition matrix element ⟨*u*_*c*_(*r*)|*p̂*(θ)|*u*_*v*_(*r*)⟩, where *u*_*c*_(*r*) and *u*_*v*_(*r*) correspond to the Bloch function of the conduction and valence bands, respectively. *p̂*(θ) is defined as ***e*** (θ)·*p̂*, where ***e*** (θ) and *p̂* represent a unit vector, whose direction is determined by θ, and the momentum operator, $-i\hbar \left(\frac{\partial }{\partial x},\frac{\partial }{\partial y}\right)$ , respectively. Figure 4c displays a 2D map of the local matrix element along with a corresponding structural model. The θ dependence, which determines polarization selectivity in optical transitions, is shown as polar plots at each location; the corresponding interlayer configurations are shown on the right. Interestingly, the θ dependence exhibits a p-like shape, except at the A, B, and C sites, indicating linearly polarized light emission at each location. However, due to the *C*_3_ symmetry of the system, the overall polarizations cancel each other out, resulting in PL without a specific linear polarization. When strain induces the *C*_3_ symmetry breaking, as evidenced by the moiré pattern distortion, the cancellation of light polarization is incomplete, resulting in a finite residual linear polarization. Moreover, the distortion of χ­(r) induced by strain can further modulate the spread of the exciton wave function, contributing to the observed residual linear polarization by affecting the emission-contributing region. Figure 4c should be regarded as a local transition-matrix analysis for finite DLP, not as an evaluation of the circular valley selection rule; see Supporting Note S2 for a more detailed explanation. For comparison, the corresponding H-type calculation is shown in Figure S7.

To clarify the mechanism for the emergence of finite linear polarization without relying on large-scale calculations, we provide a minimal symmetry-based model in Supporting Note S3. In this model, three *C*_3_-related local emission channels cancel in the symmetric limit. This cancellation argument does not require complete atomic reconstruction: any moiré structure that preserves global *C*_3_ symmetry gives zero net DLP in the model, whereas finite DLP requires *C*_3_-breaking inequivalence among the local emission channels. Strain-induced distortion of the moiré pattern and the exciton envelope is then represented by an effective *C*_3_-breaking parameter, which makes the three local emission weights inequivalent and yields a finite far-field DLP that is linear in this parameter to first order. This residual polarization can arise even from weak strain because the strain effect is amplified in moiré superlattices, as discussed above. This finding highlights the need to account for residual strain when interpreting linearly polarized PL from moiré excitons as a readout of valley coherence. To provide an independent directional test, we measured an additional hBN-encapsulated WSe_2_/WS_2_ moiré heterostructure transferred across a thickness step in the bottom hBN, thereby introducing a preferential in-plane strain axis. The small but finite DLP near the step has an emission axis approximately perpendicular to this strain axis, consistent with the uniaxial *C*_3_-breaking model; experimental details and further discussion are provided in Figure S8. The hBN-step control was prepared by dry transfer, showing that the directional response is not specific to CVD growth. The net DLP is expected to depend on the spatial coherence of the strain principal axis.

We note that this conclusion does not imply that weak *C*_3_ symmetry breaking immediately destroys circularly polarized valley readout. Circular polarization primarily probes the population imbalance between the K and −K valleys, whereas linear polarization probes their coherent superposition. A weak *C*_3_-breaking perturbation can generate an additional linear-polarization component through incomplete cancellation of local emission channels, even when the circularly polarized contrast associated with valley polarization remains approximately valid. Actually, helicity-resolved PL and Raman mapping yield a Pearson correlation coefficient of r = 0.0416 between DCP and the WS_2_ A_1_′ Raman frequency (Figure S9). A simple discussion of this distinction is provided in Supporting Note S4. The present result should thus be regarded as a caution specifically for interpreting finite DLP as evidence of valley coherence in moiré excitons.

In conclusion, our polarization-resolved PL and Raman mapping of WSe_2_/WS_2_ moiré superlattices show that finite PL DLP from moiré excitons should not be interpreted simply as a signature of valley coherence. Unlike monolayer WSe_2_, where the PL linear-polarization axis follows the excitation linear polarization through valley-coherent optical selection rules, the moiré heterostructure exhibits a finite DLP whose polarization axis is largely insensitive to the excitation polarization. Spatially resolved PL and Raman mapping, combined with correlation analysis, reveal that the DLP strongly correlates with Raman shifts and moiré-exciton observables, suggesting that unintentional strain is the dominant experimental correlate. We attribute this behavior to strain-amplified distortion of the moiré atomic registry and moiré potential landscape, which modifies the relative weights of local emission channels and the spatial distribution of the exciton envelope. These results show that even weak residual strain can generate valley-coherence-independent linear polarization in moiré exciton emission. Thus, residual strain and moiré-amplified symmetry breaking must be considered when using linearly polarized PL as an optical readout of valley coherence in TMD moiré heterostructures.

## Supplementary Material



## Data Availability

The data supporting the plots in this paper and the findings of this study are available from the corresponding author upon request.

## Notes

Sample preparation and optical measurement procedures; polarization-resolved SHG; peak-decomposition analysis; drift-correction procedure; carrier-corrected Raman analysis; regression results; AFM characterization; H-type transition-matrix calculation; controlled-strain and helicity-resolved PL measurements; and supporting symmetry-based models (PDF)
