# Supplementary material for: Strain Correlated Linearly Polarized Photoluminescence in WSe2/WS2 Moiré Superlattices
Source: Nano Lett. 2026 Sep 23;26(39):13255–62. doi: 10.1021/acs.nanolett.6c02363 (PMC13651620; doi:10.1021/acs.nanolett.6c02363)
Supplement: Supplementary file 1 [file nl6c02363_si_001.pdf]

Supplementary materials

Strain Correlated Linearly Polarized Photoluminescence in WS_2/WSe_2 Moiré Superlattices

Yuto Urano[1,2], Ryo Tamura[3,4], Yui Tamogami[1,5], Toshikaze Kariyado[1], Yasumitsu Miyata[1], Daichi Kozawa[1], Kenji Watanabe[6], Takashi Taniguchi[1], and Ryo Kitaura[1,2,]*

[1] Research Center for Materials Nanoarchitectonics, National Institute for Materials Science, 1-1 Namiki, Tsukuba, Ibaraki 305-0044, Japan

[2] Graduate School of Chemical Sciences and Engineering, Hokkaido University, 5, Kita 8 Nishi, Kita-ku, Sapporo, Hokkaido 060-8628, Japan

[3] Center for Basic Research on Materials, National Institute for Materials Science, 1-1 Namiki, Tsukuba, Ibaraki 305-0044, Japan

[4] Graduate School of Frontier Sciences, The University of Tokyo, 5-1-5 Kashiwanoha, Kashiwa, Chiba 277-8568, Japan

[5] Department of Physics, Tokyo Metropolitan University, 1-1 Minamiosawa, Hachioji, Tokyo 192-0397, Japan

[6] Research Center for Electronic and Optical Materials, National Institute for Materials Science, 1-1 Namiki, Tsukuba, Ibaraki 305-0044, Japan

* Correspondence to KITAURA.Ryo@nims.go.jp

Methods

Preparation of WSe_2/WS_2 moiré heterostructures

hBN flakes were mechanically exfoliated onto SiO_2/Si substrates with a 285-nm-thick SiO_2 layer. Monolayer WS_2 was first grown on the hBN flakes by powder CVD using WO_3 powder, KBr powder as a growth promoter, and sulfur flakes as precursors. The growth was performed under N_2 flow at 600 sccm. The substrate and WO_3 source were heated to 970 °C over 40-50 min, while the sulfur source was heated to 180 °C for 10 min when the substrate temperature reached 950 °C. After growth, the system was rapidly cooled with an electric fan. WSe_2 was subsequently grown on the as-grown WS_2/hBN sample by MOCVD using $(t\text{-}BuN=)_2W(NMe_2)_2$ and $(t\text{-}C_2H_5)_2Se_2$ as W and Se precursors, respectively. The sample was first heated to 800 °C in an N_2/H_2 flow of 679/21 sccm and annealed for 10 min to remove surface impurities. After cooling to 100 °C, the sample was reheated to 700 °C, and the precursors were supplied for 10 min by N_2 bubbling under atmospheric pressure. The precursor reservoirs were kept at room temperature during growth. After growth, the system was rapidly cooled with an electric fan. Finally, the directly grown WSe_2/WS_2 heterostructure was encapsulated with a top hBN flake.

Optical measurements

Optical responses were measured using a home-built microspectroscopy system. PL measurements were performed using a Ti:sapphire pulsed laser (Mai Tai HP, Spectra-Physics) operating at 740 nm, with a repetition rate of 80 MHz and a pulse width of 100 fs. The laser beam was focused to a spot approximately 1 μm in diameter. A typical excitation power of ~100 nW was used. Raman spectroscopy experiments were performed using a He-Ne continuous-wave laser (HNL050L, Thorlabs) at 632.8 nm, with a focused spot size of approximately 1 μm in diameter, similar to the pulsed laser setup. For both Raman and PL observations, we used a liquid-nitrogen-cooled CCD detector (PyLoN 400, Teledyne Princeton Instruments) and a spectrometer (IsoPlane 320, Teledyne Princeton Instruments). The sample was mounted in an ultra-low vibration cryogen-free optical cryostat (CryoAdvance 50, Montana Instruments) and maintained at a temperature of 3.5 K. Linear polarization was generated and detected using polarizers (GTH10M-A for excitation and LPNIR100-MP2 for detection, Thorlabs) as well as λ/2 plates (AHWP05M-580 for excitation and AHWP05M-980 for detection, Thorlabs) located on both excitation and detection sides. These optical elements were controlled by automated rotation stages, and all measurements were conducted fully automatically through a homemade LabVIEW program.

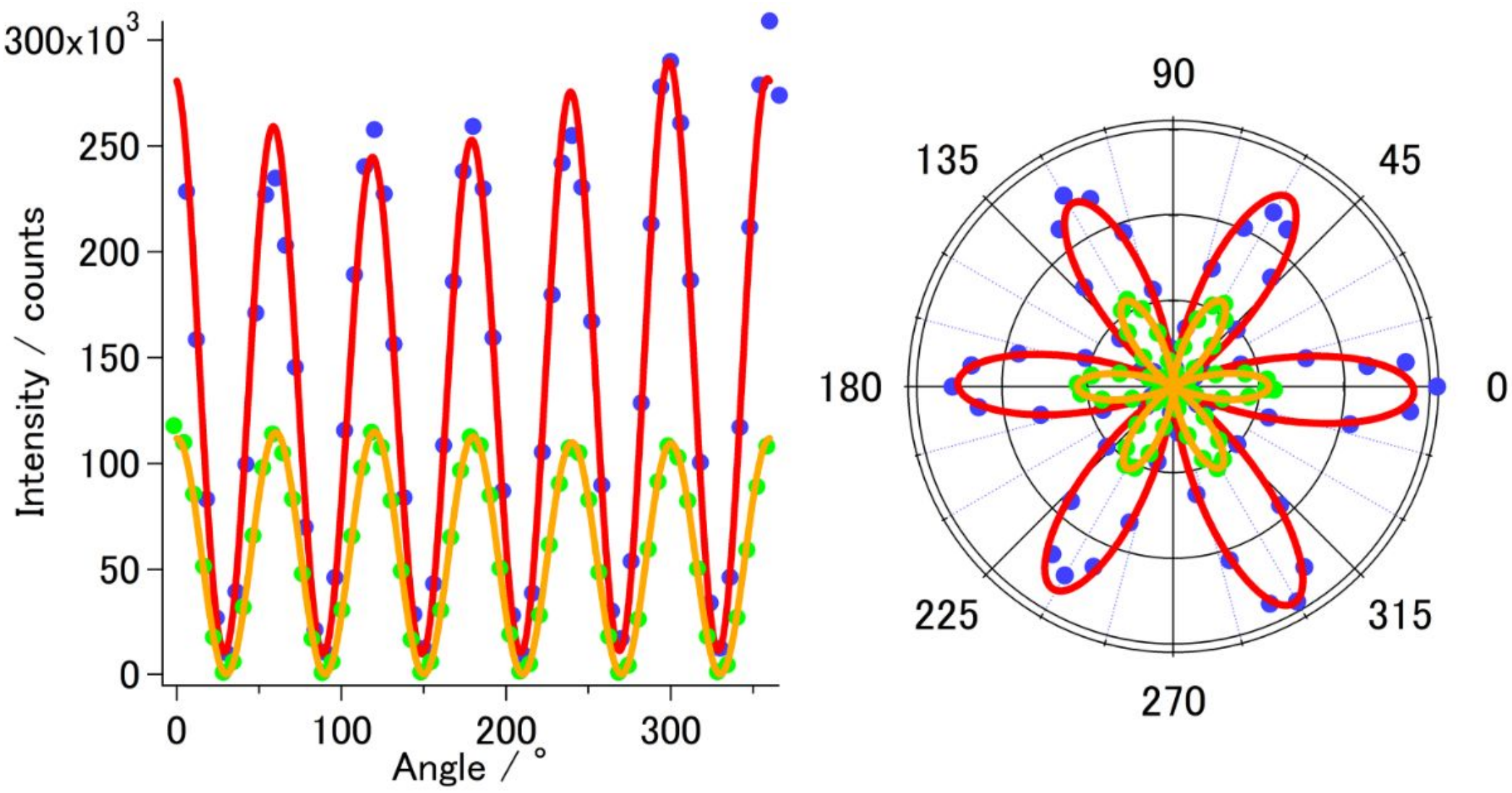

Figure S1. Polarization-resolved SHG of the measured WSe_2/WS_2 heterobilayer and a reference monolayer WSe_2 region acquired under identical excitation and collection conditions. Left and right show the angular dependences in Cartesian and polar representations, respectively. Blue and green points are the measured SHG intensities of the heterobilayer and monolayer reference, respectively; red and orange curves are fits to the corresponding sixfold responses. The markedly stronger heterobilayer response supports constructive addition of the layer SH fields for near-0° R-type stacking. The heterobilayer fit establishes the crystallographic reference frame and places the zigzag direction at $0.16 \pm 0.20°$ relative to the laboratory horizontal axis.

Direct van der Waals epitaxy of TMD heterobilayers preferentially produces rotationally commensurate high-symmetry configurations near 0° and 60°, rather than a broad distribution of arbitrary twist angles. Directly grown WSe_2/WS_2 and related TMD heterobilayers have been reported in both parallel R-type and antiparallel H-type configurations (Hsu et al., Nat. Commun. 2018; Yuan et al., Nat. Mater. 2020). Growth statistics therefore restrict the principal structural candidates but do not by themselves determine the stacking of the measured region.

The sixfold SHG angular dependence in Fig. S1 establishes the relative crystallographic orientation modulo 60° and therefore does not by itself distinguish R- and H-type stacking. We therefore combine the angular response with a sample-specific intensity comparison under identical excitation and collection conditions. Figure S1 includes a reference monolayer WSe_2 trace acquired under the same conditions; the markedly stronger heterobilayer response is consistent with constructive interference between the layer SH fields for near-0° R-type stacking, whereas near-60° H-type stacking would give destructive interference and a substantially weaker heterobilayer signal (Hsu et al., ACS Nano 2014; Hsu et al., Nat. Commun. 2018; Sun et al., Nature 2022). We therefore assign the measured heterobilayer as near-0° R-type. This assignment

identifies the high-symmetry stacking class but does not determine the local atomic registry with mathematical precision.

The 0° and 90° polarization labels used in the PL experiment denote horizontal and vertical laboratory-frame axes with respect to the optical bench; they are not crystallographic labels. The moiré-exciton emission axis varies approximately from 36° to 46° in the same frame and is not interpreted as universally locked to a crystallographic direction. In the present model, the DLP axis is selected primarily by the local principal axis of the C_3-breaking field and the sign of the strain–polarization coupling.

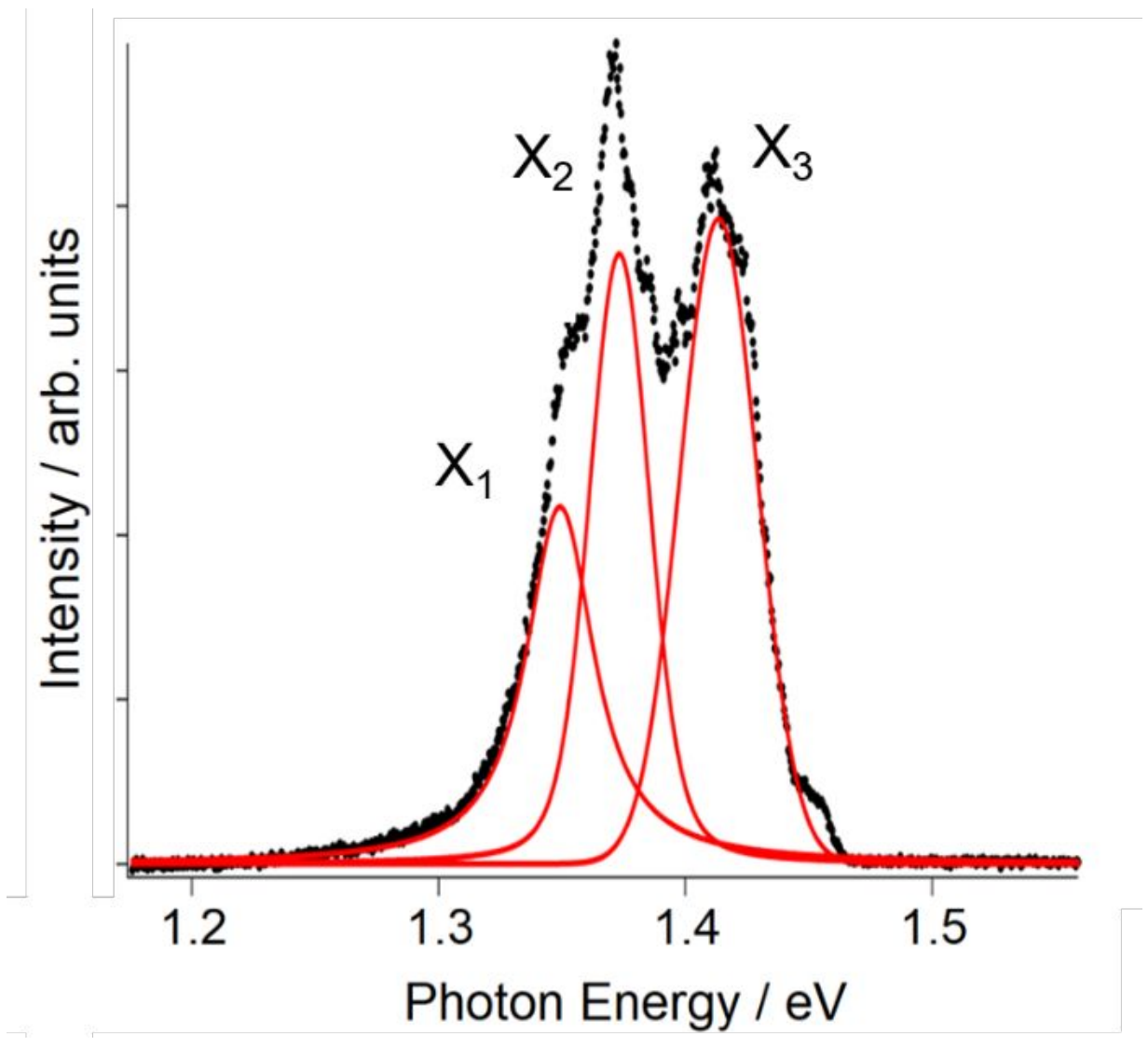

Figure S2. Peak decomposition of analyses and peak assignment of the low-energy moiré-exciton resonance shown in Fig. 1(d)

As shown in the Figure, the low-temperature PL spectrum contains three low-energy resonances, X_1, X_2, and X_3, in the interlayer-exciton range for near-aligned WSe_2/WS_2 heterobilayers. Their cryogenic appearance, separation from the intralayer excitons, and agreement with comparable heterobilayers support their assignment to the interlayer moiré-exciton emissions. Reported microscopic interpretations of such multipeak spectra include (i) an on-site dipolar occupation ladder (Park et al.; Tamogami et al.), (ii) ground and excited center-of-mass levels in a moiré potential well (Tran et al.; Chatterjee et al.), and (iii) spin-, valley-, moiré-quasi-angular-momentum-, registry-, or conduction-band-spin-resolved branches (Jin et al.; Rossi et al.). Our fixed-power, ungated, zero-field spectrum cannot distinguish these possibilities.

For the present sample class, the occupation-ladder picture is the most plausible working interpretation, but not a unique assignment. The closest precedent is the power-dependent study by Tamogami et al. of directly grown, hBN-supported, near-0° WSe_2/WS_2; Park et al. independently established density-dependent dipole ladders in R-stacked WSe_2/WS_2. In this picture, X_1 is the lowest single interlayer exciton, with the electron in WS_2, the hole in WSe_2, and center-of-mass localization near the Rₕ^{x} minimum, whereas X_2 is an interaction-shifted on-site two-exciton state. We leave X_3 unassigned because higher rungs can contain additional spin and orbital structure (Cho et al.). Thus, the secure assignment used here is the collective interlayer moiré-exciton emission manifold. The remaining peak-level ambiguity does not affect the fixed-axis DLP, its excitation-polarization independence, or its Raman correlation, and therefore does not alter our conclusions.

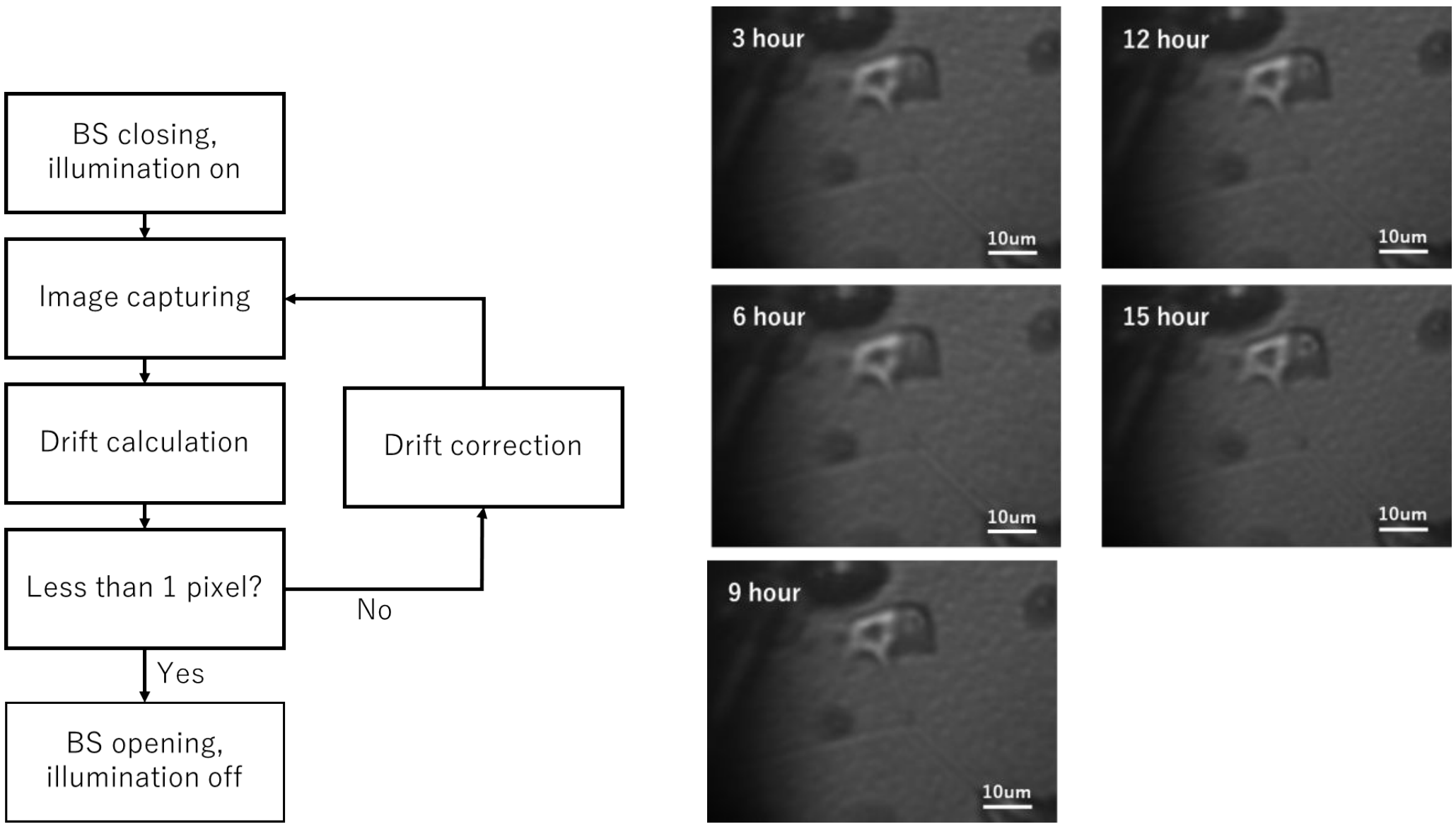

Figure S3. (Left) Block diagram illustrating the drift-correction procedure. BS stands for beam shutter. (Right) Series of optical images acquired during a mapping measurement.

Our microspectroscopy system is stable, with a typical spatial drift of less than 1 μm over a three-hour period. However, since mapping measurements can take several days, it is crucial to account for spatial drift to ensure the resulting spectral maps are accurate and meaningful. To correct this drift, we capture an optical image after each mapping line and calculate the drift using an enhanced correlation method. The calculated drift is measured in pixels and converted into pulses for the stepping motor to perform the correction. All processes—such as closing the beam shutter to cut the excitation laser beam, turning illumination on, capturing an image, calculating the drift, correcting the drift, capturing an additional image to verify if the correction worked, turning illumination off, and reopening the shutter—can be automated using a LabVIEW VI. The correction process is shown in the block diagram in Fig. S3 (left), ensuring drift correction with 1-pixel accuracy. Additionally, we incorporate focus correction into the drift-correction LabVIEW VI. For focus correction, we direct the excitation laser reflected from the sample through a 50-μm pinhole using a 15-cm lens. The light intensity passing through the pinhole is then measured with a photodiode. Focus correction involves adjusting the sample height to maximize light intensity. This focus correction is performed before drift correction to ensure that the drift correction can be carried out effectively. The figures show optical images of samples collected during a mapping measurement. As seen in the figures, there is almost no shift across all images after the drift correction. This allows us to conduct detailed mapping measurements to analyze the relationship between linear polarization and different quantities.

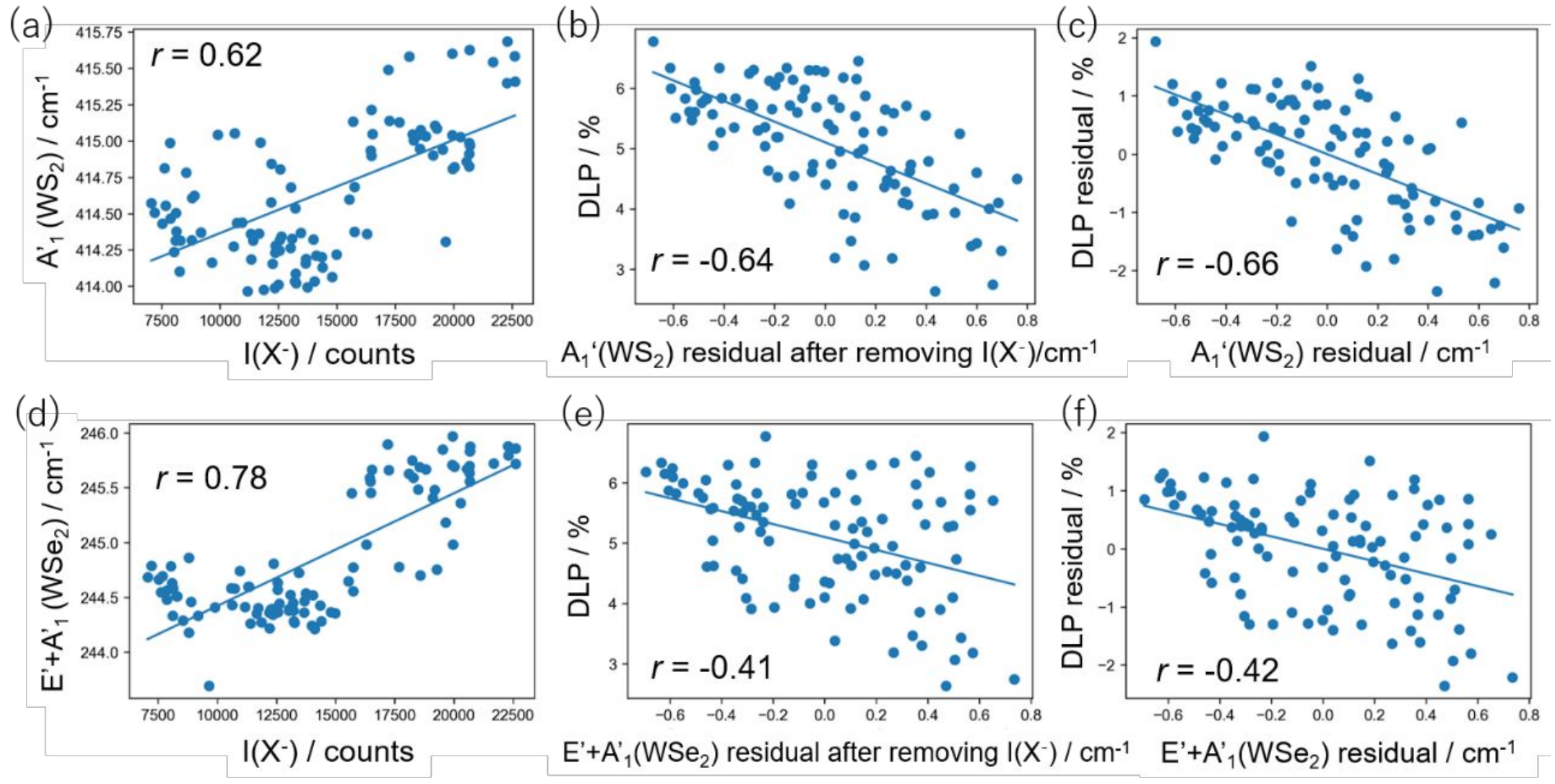

Figure S4. Carrier-corrected Raman residual analysis. (a,d) Correlations between the Raman descriptors and the WSe_2 intralayer trion intensity I(X-), used as an experimental proxy for local carrier-density or Fermi-level variations. The descriptors are the WS_2 A_1' mode (often denoted A_1g in multilayer notation) in (a) and the unresolved WSe_2 $E'+A_1'$ feature near 250 cm^{-1} in (d). (b,e) Correlations between DLP and the carrier-corrected Raman residuals obtained after subtracting the component linearly correlated with I(X-). (c,f) Partial-correlation analysis after linearly removing the I(X-) contribution from both DLP and the Raman descriptor.

The WS_2 and WSe_2 Raman descriptors correlate with $I(X^-)$ with r = 0.623 and 0.780, respectively, confirming that carrier-density variation contributes appreciably to the raw Raman maps. Nevertheless, the DLP-Raman relationship persists after this component is removed. The raw DLP correlations are r = -0.645 for WS_2 and r = -0.438 for WSe_2. After removal of the $I(X^-)$-correlated Raman component, the corresponding values remain r = -0.639 and r = -0.410; the partial correlations obtained after correcting both quantities are r = -0.657 and r = -0.422. By comparison, the direct DLP-$I(X^-)$ correlation is substantially weaker, r = -0.233. Thus, carrier density contributes to the Raman variation but does not account for the principal DLP-Raman relationship. We therefore refer to the remaining carrier-independent Raman component conservatively as a strain-like lattice-distortion descriptor rather than as a unique quantitative strain measurement. The approximately 250 cm^{-1} WSe_2 feature is treated as an unresolved $E'+A_1'$ descriptor because these modes are nearly degenerate under the present measurement conditions. The carrier-corrected WS_2 A_1' residual has $\sigma = 0.355$ cm^{-1} and a central 90% interval of −0.538 to +0.601 cm^{-1}. Using the reported biaxial-strain coefficient of approximately −1.8 cm^{-1}/% (Michail et al., 2023; Waheed et al., 2025) gives a relative strain-equivalent scale of $\sigma \approx 0.20\%$ and −0.33% to +0.30% about the fitted mean. Because A_1' is also doping-sensitive and a scalar

Raman shift does not resolve the deviatoric strain component that breaks C_3, this is only an order-of-magnitude estimate, not an absolute or tensor-resolved strain map. For comparison, 0.1% physical strain in WS_2 changes the calculated moiré period by approximately 2.6%; the 2% model in Fig. 4 is used only for visual clarity.

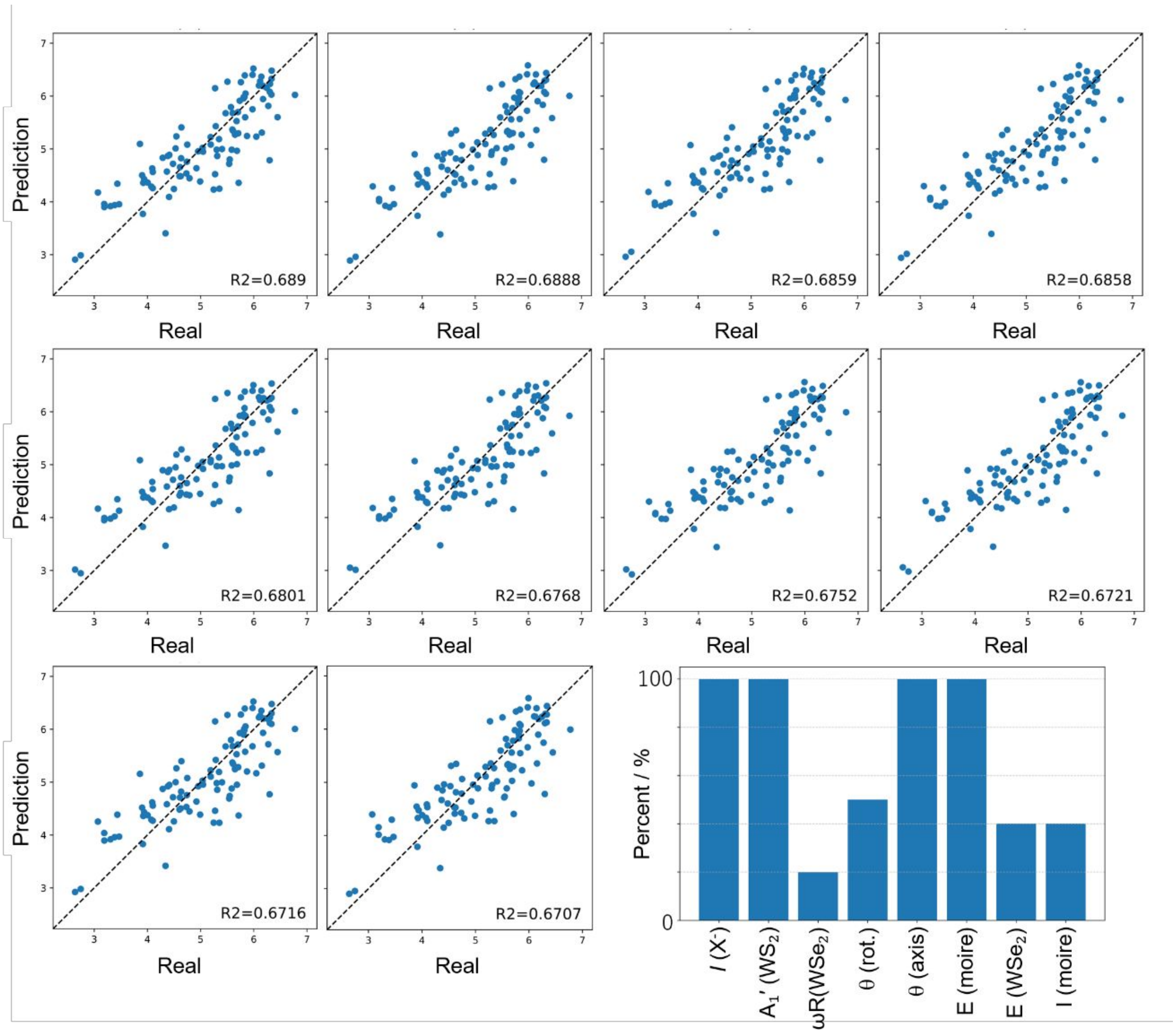

Figure S5. Relations between the observed and predicted DLP. The predictions were made using a top-10 model developed by linear regression. The bar graph shows the physical quantities used in the top-10 models. Here, $\omega R(WSe_2)$ denotes the WSe_2 Raman descriptor, defined as the peak frequency of the unresolved $E'+A_1'$ feature near $250\ cm^{-1}$.

We have developed prediction models using all combinations of the eight physical quantities, resulting in a total of 255 (=2^8 - 1) models. The plots above illustrate the ten best prediction models among these combinations. The bar graph displays the physical quantities utilized in the top ten models. Notably, $I(X^-)$, A_1' (WS_2), θ (axis), and E(moiré) were included in all ten models. This strongly suggests that these four quantities are crucial for explaining the observed DLP in the moiré sample.

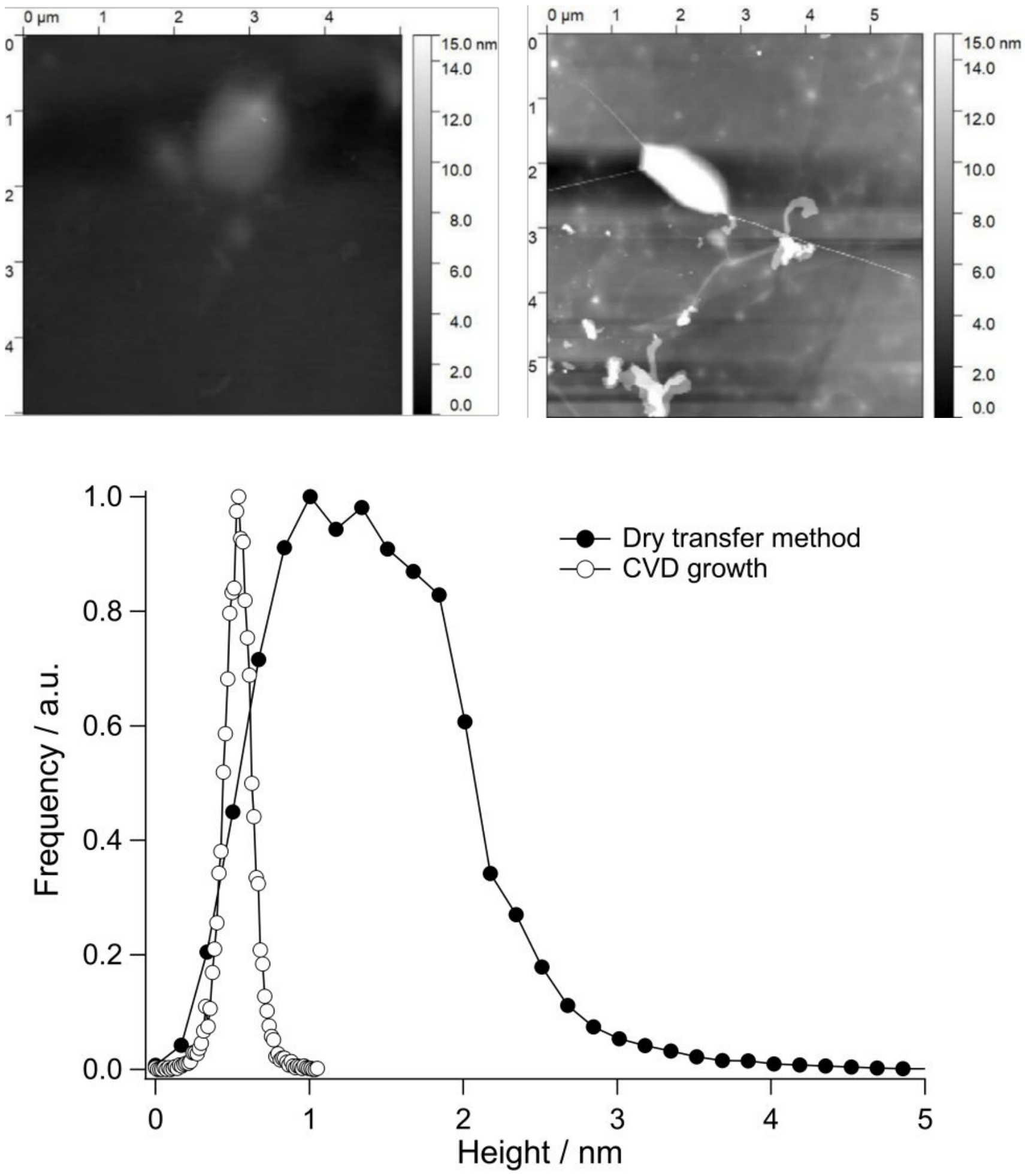

Figure S6. AFM images of (top left) the CVD-grown WSe_2/WS_2 sample and (top right) a dry-transferred sample. (Bottom) Height distributions of the CVD-grown and dry-transferred samples. These moiré samples are encapsulated between hBN flakes.

These AFM images are representative of each sample type, and the high regions correspond to bubbles trapped between layers. In the dry-transferred sample, bubbles can be introduced during each transfer step used to assemble the $hBN/WSe_2/WS_2/hBN$ stack. In the CVD-grown sample, bubbles can form when a top hBN flake is placed on the $WS_2/WSe_2/hBN$ surface to protect the heterostructure. The height distributions clearly show that, apart from the encapsulated bubbles, the surface of the CVD-grown sample remains relatively flat, consistent with the formation of a clean interface between WS_2 and WSe_2. In contrast, the dry-transferred sample exhibits a much broader height distribution, attributed to bubbles trapped between layers during transfer.

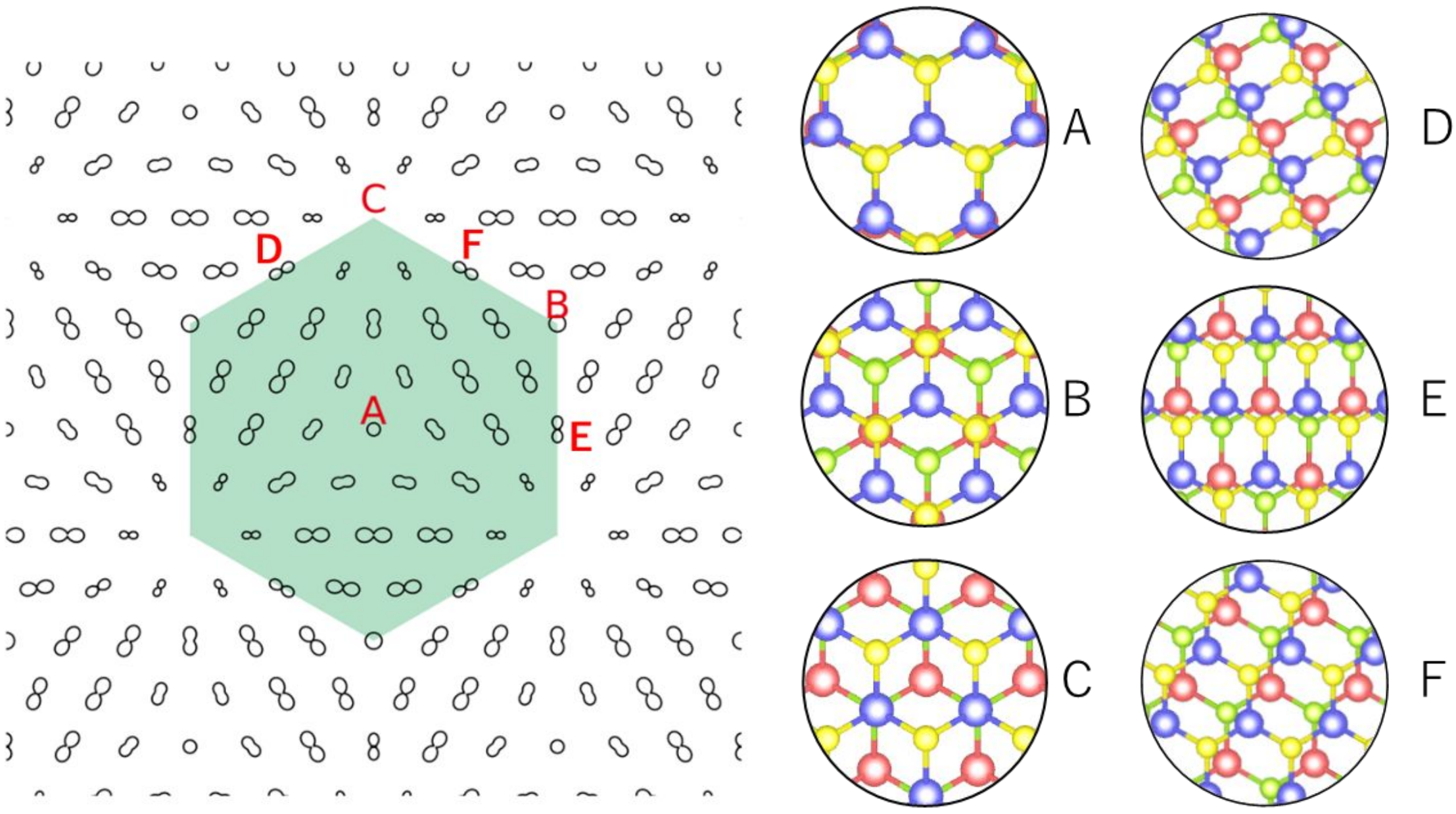

Figure S7. Distribution of the local transition matrix element in the H-type moiré lattice and the corresponding local atomic registries. The green hexagon represents the Wigner-Seitz cell of the moiré lattice, whose local structures around A-F are shown on the right. Blue, red, yellow, and green spheres denote W (top layer), W (bottom layer), Se, and S atoms, respectively. Circles and peanut-shaped figures are polar plots of the local transition matrix element. Except for locations A, B, and C, all transition matrix elements exhibit a p-orbital-like shape, suggesting possible local polarization selectivity in optical transitions.

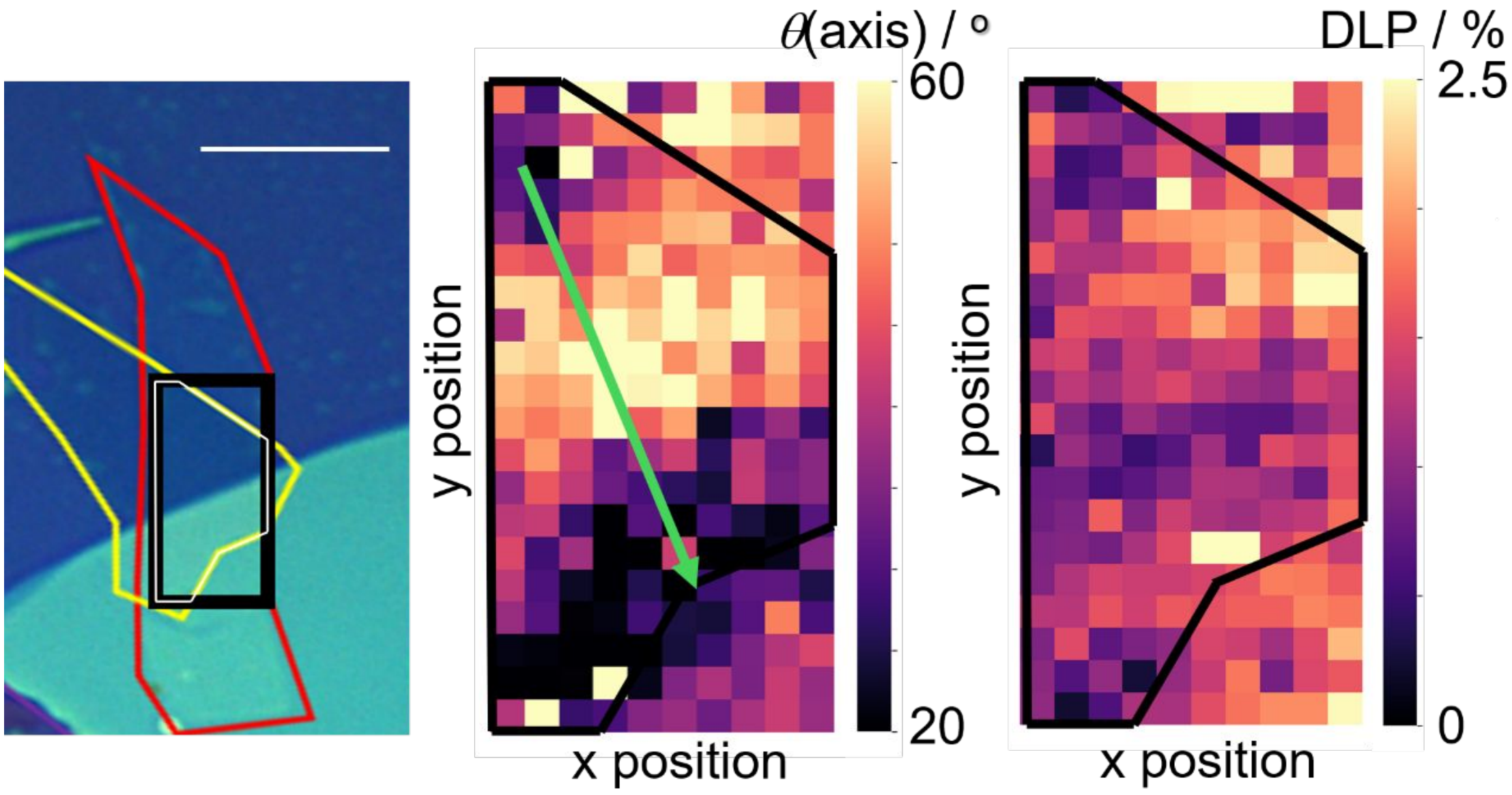

Figure S8. Polarization-resolved PL mapping of an hBN-encapsulated WSe_2/WS_2 moiré heterostructure transferred across a thickness step in the bottom hBN. Left: optical image, where the red and yellow outlines indicate the WS_2 and WSe_2 regions, respectively, and the black rectangle indicates the mapped region. Center and right: maps of the fitted polarization axis, θ(axis), and DLP, respectively. The green arrow in the center panel indicates the representative fitted PL polarization direction in the step-affected region; this direction is approximately perpendicular to the preferential in-plane strain axis introduced by the bottom-hBN step. The scale bar represents 10 μm.

The bottom-hBN step was used to introduce a preferential in-plane strain axis while retaining the encapsulated geometry required for cryogenic optical measurements. Low-temperature polarization-resolved PL was measured near the step and in a nearby flat region. The DLP is small but finite in both regions, 1.66% near the step and 1.26% in the flat region. Because the local strain magnitude is spatially inhomogeneous and is not independently calibrated, this difference is not interpreted as a quantitative DLP–strain relation. The more informative observation is directional: the fitted emission axis changes from 50.6 ± 3.2° in the flat region to 39.4 ± 3.9° near the step and is approximately perpendicular to the preferential strain axis in the step-affected region.

The minimal C_3-breaking model predicts that a uniaxial perturbation fixes the emission axis relative to the principal strain axis, with the sign of the microscopic strain–polarization coupling determining whether the response is parallel or perpendicular. The approximately perpendicular axis observed near the hBN step is therefore consistent with one of the two symmetry-allowed responses. A small net DLP can coexist with this directional alignment because spatially varying local contributions partially cancel within the approximately 1-μm optical spot.

The dry-transferred hBN-step specimen and the directly grown specimen probe complementary strain landscapes. Dry transfer can introduce bubbles, wrinkles, trapped residues, and local deformations at several interfaces, producing strain fields whose principal-axis directions vary within the optical spot and partially cancel in the far-field DLP. By contrast, the directly grown specimen has a comparatively uniform mesoscale morphology and lacks multiple transfer interfaces, yet exhibits a clear finite DLP. These observations are consistent with the net DLP depending on both the distortion amplitude and the spatial coherence of its principal axis.

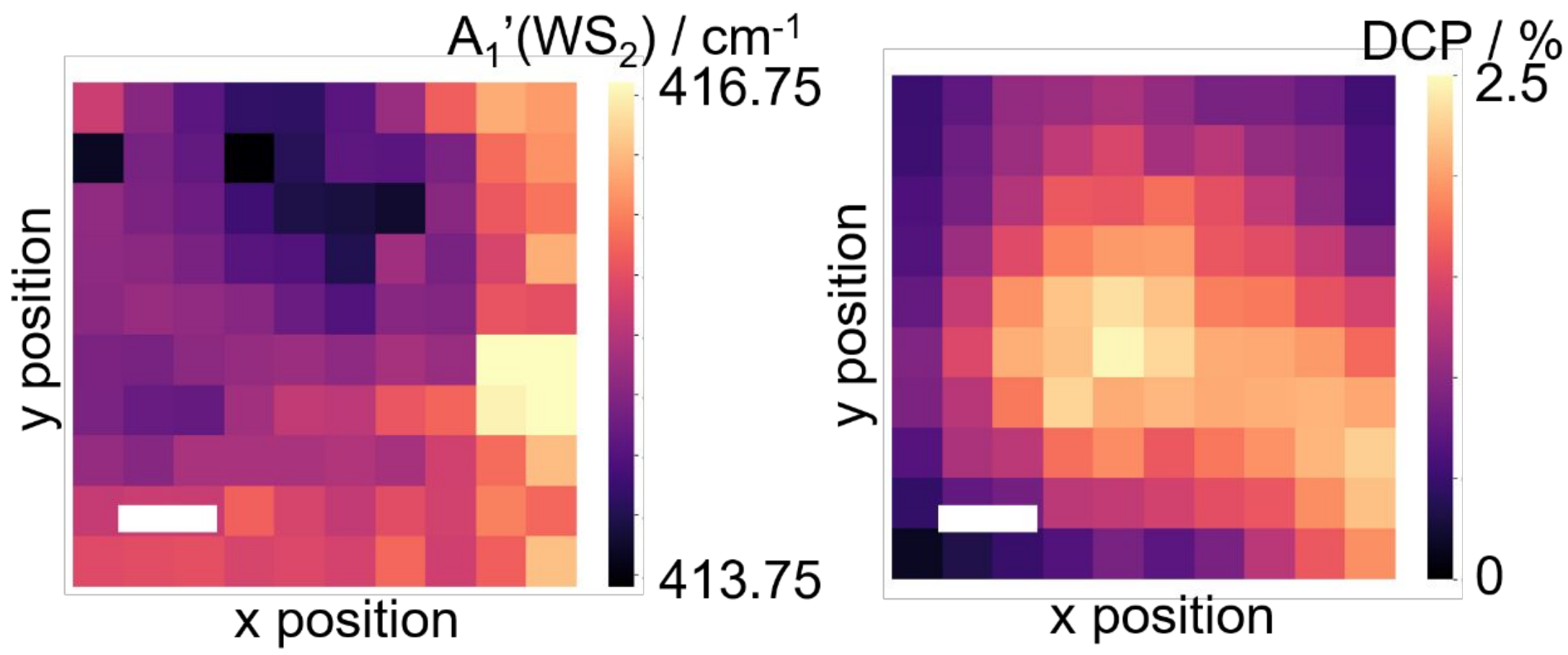

Figure S9. Spatial comparison between the WS_2 A_1' Raman frequency and the degree of circular polarization (DCP) of the moiré-exciton PL. The maps were acquired in a separate measurement sequence over approximately the same region of the heterostructure as the linear-polarization measurements, although exact pixel-for-pixel reproduction of the original field of view was not possible. Left: WS_2 A_1' Raman-frequency map. Right: DCP map evaluated over the same moiré-exciton spectral range. The two maps shown here were registered to each other for the correlation analysis. The DCP is defined as DCP = $(I_{\sigma+} - I_{\sigma-})/(I_{\sigma+} + I_{\sigma-})$. Their pixelwise Pearson correlation coefficient is r = 0.0416, indicating no appreciable linear correlation within this mapped region. Scale bars: 1 μm.

Because the field of view is not pixel-identical to that used for the DLP maps, this coefficient should not be interpreted as a direct same-pixel comparison with the DLP–Raman coefficients reported in the main text. Nevertheless, the nearly zero correlation provides complementary evidence that DCP and DLP have markedly different spatial sensitivities in the measured regime.

Supporting Note S1. Definitions and interpretation of statistical metrics

For two co-registered mapped quantities X and Y, the Pearson correlation coefficient is defined as $r_{XY} = \mathrm{cov}(X, Y)/(\sigma_X \sigma_Y)$, with $-1 \leq r_{XY} \leq 1$. Here, $\mathrm{cov}(X, Y) = \Sigma_i(X_i - \bar{X})(Y_i - \bar{Y})/(N - 1)$ is the sample covariance evaluated over the N co-registered pixels, where i indexes the pixels; $\bar{X}$ and $\bar{Y}$ are the corresponding pixelwise means, and σ_X and σ_Y are the corresponding sample standard deviations. The Pearson coefficient specifically quantifies linear correlation between X and Y. A positive sign means that the two quantities tend to increase or decrease together, whereas a negative sign means that one tends to decrease as the other increases. The magnitude $|r_{XY}|$ measures the strength of their pixelwise linear association: values closer to 1 indicate stronger linear correspondence, while values near 0 indicate little linear association. No universal numerical threshold separates weak, moderate, and strong correlation because the magnitude also depends on noise, dynamic range, nonlinearities, and spatial sampling. Pearson r is dimensionless, does not measure the strength of a microscopic coupling, and does not establish causality. Accordingly, $r \approx 0$ does not exclude a nonlinear relationship or a dependence on a common unmeasured variable. We therefore use r as a descriptive measure of linear similarity between co-registered maps.

For observed values y_i and out-of-fold predictions $\hat{y}_i$, the coefficient of determination is $R^2 = 1 - \Sigma_i(y_i - \hat{y}_i)^2/\Sigma_i(y_i - \bar{y})^2$, where i indexes the N mapped pixels, $\hat{y}_i$ is the prediction for pixel i obtained from a model trained without the validation fold containing that pixel, and $\bar{y}$ is the mean of the observed y_i values. $R^2 = 1$ corresponds to perfect prediction, $R^2 = 0$ means that the predictions perform no better than using the mean of the observed data, and $R^2 < 0$ means that they perform worse than that baseline. Unlike Pearson r, R^2 is a goodness-of-prediction metric and has no sign indicating co-variation or anti-variation; it is also not generally equal to the square of a Pearson coefficient. The present cross-validated value $R^2 = 0.689$ means that 68.9% of the pixelwise DLP variance is described by the out-of-fold predictions. It does not establish causality or the relative physical importance of individual predictors.

Supporting Note S2. Local transition-matrix analysis and circular valley selection rule

The local transition-matrix analysis shown in Fig. 4c should be distinguished from the circular valley selection rule of moiré excitons. Figure 4c evaluates the angular dependence of the local transition matrix element at different atomic registries within the moiré unit cell. This analysis is useful for visualizing local linearly polarized emission components and for discussing how these local components can cancel under C_3 symmetry or remain finite when strain makes the C_3-related emission weights inequivalent.

In contrast, the circular valley readout is governed by the global C_3 quasi-angular momentum of the moiré exciton. In a C_3-symmetric lattice, the K point remains invariant under C_3 rotation up to a reciprocal lattice vector. Consequently, valley Bloch states at K can be classified according to their C_3 eigenvalues. The circular optical selection rule arises from the conservation of C_3 quasi-angular momentum modulo 3, incorporating the C_3 transformation properties of both the electron and hole valley Bloch functions, as well as the phase structure of the exciton envelope across the moiré unit cell. Such a global symmetry property is not captured by a purely local transition-matrix map. Figure 4c, therefore, does not aim to assess whether the circularly polarized valley readout is preserved or suppressed.

The role of Fig. 4c in the present work is more specific: it illustrates that local optical transitions can contain linearly polarized components. In a C_3-symmetric moiré lattice, three C_3-related local emission channels contribute with equal weights, and their linearly polarized components cancel in the far field. When residual strain distorts the moiré atomic registry and the exciton envelope, these local weights become inequivalent. This incomplete cancellation provides a symmetry-based route to finite far-field DLP, as discussed in Supporting Note S3. The corresponding H-type calculation is shown in Fig. S7.

Supporting Note S3. Minimal C_3-breaking model for finite far-field DLP

Here, we introduce a minimal symmetry-based model to show how a finite far-field DLP can emerge from weak C_3 symmetry breaking in a moiré exciton system. This model is not intended to provide a full microscopic calculation of the exciton wavefunction or optical matrix elements. Rather, it clarifies the symmetry requirement for the incomplete cancellation of local linearly polarized emission components. We consider three local emission channels related by C_3 rotation within a moiré unit cell. Each channel is assumed to have a local linearly polarized emission component, as shown by the DFT calculation. Using the complex electric-field amplitudes along the x and y axes, E_x and E_y, the linear Stokes parameters are written as

$$Q = S_1 = \langle |E_x|^2 - |E_y|^2 \rangle,$$

$$U = S_2 = 2\,\mathrm{Re}\langle E_x E_y^* \rangle,$$

where <...> denotes time or ensemble averaging. Thus, Q represents the intensity imbalance between x- and y-polarized components, whereas U represents the intensity imbalance between +45° and −45° linear polarization components. The complex linear-polarization parameter used in the minimal model is therefore defined as

$$\mathcal{P} = Q + \mathrm{i}U = S_1 + \mathrm{i}S_2.$$

If the local polarization axis of the j-th channel is φ_j, its contribution is written as

$$\mathcal{P}_j = p_0\, w_j \exp(\mathrm{i}2\varphi_j)$$

Here, p_0 is the local degree of linear polarization, and w_j is the effective emission weight of the j-th channel. The factor of 2 in $\exp(\mathrm{i}2\varphi_j)$ reflects the fact that a linear polarization axis is unchanged under $\varphi_j \rightarrow \varphi_j + \pi$. In the far-field DLP measurements, the sum of each contribution is observed.

$$\mathcal{P} = p_0 \Sigma_j\, w_j \exp(\mathrm{i}2\varphi_j)$$

Below, we calculate $\mathcal{P}$ for both the C_3-symmetric case and the case with weakly broken C_3 symmetry.

1. The C_3-symmetric case

The three local polarization axes are related by 120° rotations,

$$\varphi_j = \varphi_0 + 2\pi(j-1)/3,\ j = 1, 2, 3,$$

and the three channels have identical weights,

$$w_1 = w_2 = w_3 = w_0$$

The total far-field linear polarization is then

$$\mathcal{P} = p_0\, w_0\, \Sigma_j \exp(\mathrm{i}2\varphi_j) = 0.$$

Thus, even if each local channel has a finite linearly polarized component, the far-field DLP vanishes in the C_3-symmetric limit because the three contributions cancel exactly.

2. The case with weakly broken C_3 symmetry

We introduce weak C_3 symmetry breaking through an effective parameter ε. Physically, ε represents the strain-induced inequivalence of the three local emission channels, causing inequivalence in w_j. This inequivalence can arise from distortion of the moiré pattern, modification of the local optical matrix elements, and/or distortion of the exciton envelope function. Specifically, w_j can be written as follows.

$$w_j(\varepsilon) = \int_{\Omega j(\varepsilon)} d^2r\, |\chi_\varepsilon(r)|^2\, g_j(r, \varepsilon),$$

where $\Omega_j(\varepsilon)$ is the emission-contributing region associated with the j-th local channel, $\chi_\varepsilon(r)$ is the strain-distorted exciton envelope function, and $g_j(r, \varepsilon)$ represents the local oscillator strength. In the absence of strain, C_3 symmetry makes the three weights equivalent. When strain distorts the moiré pattern, the regions $\Omega_j(\varepsilon)$, the envelope distribution $|\chi_\varepsilon(r)|^2$, and the local oscillator strength can become inequivalent among the three channels. All of these effects are phenomenologically captured by the effective C_3-breaking parameter ε.

We assume that the effective emission weight of each local channel responds linearly to the anisotropic component of the strain tensor. Let $\mathbf{e}_j = (\cos\varphi_j, \sin\varphi_j)$ be the unit vector along the local emission axis of the j-th channel. We write

$$w_j = w_0\, [1 + \lambda\, \mathbf{e}_j^T \mathbf{u}_{dev} \boldsymbol{e}_j],$$

where $\mathbf{u}_{dev}$ is the deviatoric component of the in-plane strain tensor, and λ is a phenomenological coupling constant. The hydrostatic component of strain changes all three weights equally and therefore does not generate a net far-field DLP to first order.

For a uniaxial distortion with principal axis ψ_s, the deviatoric strain tensor can be written as

$$\mathbf{u}_{dev} = u_s \begin{pmatrix} \cos 2\psi_s & \sin 2\psi_s \\ \sin 2\psi_s & -\cos 2\psi_s \end{pmatrix}$$

where $u_s = (u_{parallel} - u_{perpendicular})/2$ is the magnitude of the anisotropic strain component. This gives

$$\mathbf{e}_j^T \mathbf{u}_{dev} \boldsymbol{e}_j = u_s \cos 2(\varphi_j - \psi_s).$$

Therefore,

$$w_j = w_0\, [1 + \lambda u_s \cos 2(\varphi_j - \psi_s)].$$

This form expresses the physical assumption that local emission channels aligned parallel and perpendicular to the strain-induced distortion acquire different effective emission weights. Equivalently, by defining $\varepsilon = \lambda u_s/2$, we obtain

$$w_j = w_0\, [1 + 2\varepsilon \cos 2(\varphi_j - \psi_s)].$$

The total polarization thus becomes

$$\mathcal{P} = p_0\, \Sigma_j\, w_j \exp(i2\varphi_j).$$

The C_3-symmetric part again cancels. Keeping only the term linear in ε gives

$$\mathcal{P} = 3\, p_0\, w_0\, \varepsilon \exp(i2\psi_s).$$

The total emission intensity is

$$S_0 = \Sigma_j\, w_j = 3w_0 + O(\varepsilon^2).$$

Since $S_0 = 3w_0 + O(\varepsilon^2)$, the denominator does not modify the leading linear dependence, and we obtain the far-field DLP as

$$\mathrm{DLP} = |\mathcal{P}|/S_0 = p_0|\varepsilon| + O(\varepsilon^2).$$

This result shows that finite DLP appears linearly with the effective C_3-breaking parameter. Therefore, a weak perturbation that makes the three C_3-related local emission weights inequivalent is sufficient to generate a finite far-field DLP.

The effective channel weights w_j can be interpreted as a coarse-grained representation of the spatially integrated emission contribution. This point can be seen from a continuous expression for the complex linear-polarization component,

$$\mathcal{P}(\varepsilon) = \int_{\mathrm{cell}} \mathrm{d}^2r\, |\chi_\varepsilon(r)|^2\, m_\varepsilon(r),$$

where $\chi_\varepsilon(r)$ is the strain-dependent exciton envelope function and $m_\varepsilon(r)$ is the local complex linear-polarization density, including the local optical matrix element and polarization axis. For weak C_3 breaking,

$$|\chi_\varepsilon(r)|^2 = |\chi_0(r)|^2 + \varepsilon\, \delta\rho_\chi(r),$$

$$m_\varepsilon(r) = m_0(r) + \varepsilon\, \delta m(r).$$

We define $\rho_\chi(r) = |\chi_\varepsilon(r)|^2$ as the exciton-envelope probability density, and $\delta\rho_\chi(r)$ represents the first-order change in the spatial weighting of the exciton envelope.

Substitution gives

$$\mathcal{P}(\varepsilon) = \int_{\mathrm{cell}} \mathrm{d}^2r\, |\chi_0(r)|^2\, m_0(r) + \varepsilon \int_{\mathrm{cell}} \mathrm{d}^2r\big(\, \delta\rho\chi(r) m_0(r) + |\chi_0(r)|^2 \delta \mathrm{m}(\mathrm{r})\,\big) + O(\varepsilon^2).$$

The first term vanishes in the C_3-symmetric limit. The two linear terms show that finite far-field DLP can arise either from strain-induced distortion of the exciton envelope, which changes the spatial weighting of local emission components, or from strain-induced modification of the local optical matrix element. Thus, the phenomenological weight modulation used in the three-channel model captures both effects at the lowest order in the effective C_3-breaking parameter.

This minimal model shows that strain does not need to create a completely new optical selection rule. It only makes the three C_3-related local emission channels inequivalent. Once this happens, the local linearly polarized components no longer cancel completely, and a finite far-field DLP appears. Since small lattice strain can be amplified into a larger distortion of the moiré pattern, even weak residual strain can generate a measurable DLP in moiré exciton emission.

Relation between the effective parameter and physical strain

The parameter ε in this model is an effective optical C_3-breaking parameter and should not be identified directly with a particular component of the physical lattice-strain tensor. It contains the response of the moiré geometry, the exciton envelope, and the local optical matrix elements through an unknown strain-to-optical-weight coupling. Consequently, the model establishes the leading symmetry relation $\mathrm{DLP} = p_0|\varepsilon| + \mathrm{O}(\varepsilon^2)$, but it does not provide a parameter-free prediction of the absolute DLP. The measured DLP of 3-7% constrains only the product $p_0|\varepsilon| = 0.03\text{-}0.07$. A

quantitative conversion from physical strain to DLP would require a microscopic calculation of the strain-dependent moiré-exciton envelope and optical matrix elements.

Supporting Note S4. Weak C_3-breaking and VDF

Weak C_3 symmetry breaking can affect linear- and circular-polarization readout in qualitatively different ways. In the valley basis, circularly polarized emission is mainly associated with a valley population imbalance, whereas linearly polarized emission is associated with the coherent superposition of the K and −K valleys. Therefore, an additional strain-induced linear-polarization channel can appear without necessarily eliminating the circular contrast used to read out valley polarization. Phenomenologically, the optical dipole of a K-valley exciton can be written as

$$\boldsymbol{d}_K = d_0(\mathbf{e}_+ + \eta\mathbf{e}_-),$$

where $\mathbf{e}_+$ and $\mathbf{e}_-$ denote right- and left-circular polarization components, and η is a small helicity-mixing parameter induced by symmetry breaking. The phenomenological dipole expression $\boldsymbol{d}_K$ is directly related to the perturbative optical matrix elements. We define the transition dipole vector of a K-valley exciton as

$$\boldsymbol{d}_K = \langle G|\boldsymbol{d}|X_K\rangle,$$

where $\boldsymbol{d}$ is the electric dipole operator. Its projections onto the circular polarization basis give the optical matrix elements,

$$M_\sigma = \langle G|\mathbf{e}_\sigma^*\cdot\boldsymbol{d}|X_K\rangle = \mathbf{e}_\sigma^*\cdot\boldsymbol{d}_K.$$

Thus, $\boldsymbol{d}_K$ can be expanded as

$$\boldsymbol{d}_K = M_+\mathbf{e}_+ + M_-\mathbf{e}_-.$$

In the ideal C_3-symmetric limit, the K-valley exciton couples only to one circular helicity, so that

$$M_+^{(0)} = d_0,\ \mathrm{M}_-^{(0)} = 0.$$

When weak C_3-symmetry breaking is introduced through $H = H_0 + \lambda V$, where λV represents a weak C_3-breaking perturbation. We denote the total first-order correction to the optical matrix element by δM_σ, including corrections from the perturbed exciton state, the ground state, and, if necessary, the optical transition operator. The allowed matrix element thus becomes

$$M_+ = d_0 + \lambda\delta M_+ + O(\lambda^2),$$

whereas the previously forbidden opposite-helicity matrix element becomes

$$M_- = \lambda\delta M_- + O(\lambda^2).$$

Therefore,

$$\boldsymbol{d}_K = (d_0 + \lambda\delta M_+)\mathbf{e}_+ + \lambda\delta M_-\mathbf{e}_- + O(\lambda^2).$$

By absorbing the small correction to the allowed component into the definition of d_0 and defining

$$\eta = M_-/M_+ = \lambda\delta M_-/d_0 + O(\lambda^2),$$

we obtain the compact phenomenological form

$$\boldsymbol{d}_K = d_0(\mathbf{e}_+ + \eta\mathbf{e}_-).$$

Thus, η is the ratio of the weakly allowed opposite-helicity amplitude to the dominant allowed helicity amplitude. Fermi's golden rule then gives $I_+ \propto |d_0|^2$ and $I_- \propto |d_0|^2|\eta|^2$. The leakage into the opposite circular polarization, therefore, appears only at order $|\eta|^2$, even though the forbidden

optical matrix element itself is generated at first order in the symmetry-breaking perturbation. Specifically, the degree of circular polarization (DCP) can be written as

$$\mathrm{DCP} = (I_+ - I_-)/(I_+ + I_-) \simeq 1 - 2|\eta|^2$$

Accordingly, weak residual symmetry breaking can produce a measurable DLP while circular valley readout remains approximately intact. In this regime, the two polarization observables probe distinct aspects of the exciton state: DLP is sensitive to in-plane rotational-symmetry breaking, whereas DCP primarily reflects valley-population imbalance. A finite DLP alone therefore does not establish optically generated valley coherence.

References

1. Hsu, W.-T.; Lu, L.-S.; Wu, P.-H.; et al. Negative Circular Polarization Emissions from $WSe_2/MoSe_2$ Commensurate Heterobilayers. Nat. Commun. 2018, 9, 1356. https://doi.org/10.1038/s41467-018-03869-7.
2. Yuan, L.; Zheng, B.; Kunstmann, J.; et al. Twist-Angle-Dependent Interlayer Exciton Diffusion in WS_2-WSe_2 Heterobilayers. Nat. Mater. 2020, 19, 617–623. https://doi.org/10.1038/s41563-020-0670-3.
3. Hsu, W.-T.; Zhao, Z.-A.; Li, L.-J.; et al. Second Harmonic Generation from Artificially Stacked Transition Metal Dichalcogenide Twisted Bilayers. ACS Nano 2014, 8, 2951–2958. https://doi.org/10.1021/nn500228r.
4. Sun, X.; Zhu, Y.; Qin, H.; et al. Enhanced Interactions of Interlayer Excitons in Free-Standing Heterobilayers. Nature 2022, 610, 478–484. https://doi.org/10.1038/s41586-022-05193-z.
5. Park, H.; Zhu, J.; Wang, X.; et al. Dipole Ladders with Large Hubbard Interaction in a Moiré Exciton Lattice. Nat. Phys. 2023, 19, 1286–1292. https://doi.org/10.1038/s41567-023-02077-5.
6. Tamogami, Y.; Zhang, W.; Sakakibara, R.; Urano, Y.; Nayak, S. K.; Nakanishi, Y.; Watanabe, K.; Taniguchi, T.; Kitaura, R.; Miyata, Y. Improved Optical Uniformity of WSe_2/WS_2 Moiré Superlattices Grown on hBN toward Quantum Photonics. ACS Appl. Nano Mater. 2026, 9, 8572–8578. https://doi.org/10.1021/acsanm.6c00104.
7. Tran, K.; Moody, G.; Wu, F.; et al. Evidence for Moiré Excitons in van der Waals Heterostructures. Nature 2019, 567, 71–75. https://doi.org/10.1038/s41586-019-0975-z.
8. Chatterjee, S.; Dandu, M.; Dasika, P.; et al. Harmonic to Anharmonic Tuning of Moiré Potential Leading to Unconventional Stark Effect and Giant Dipolar Repulsion in WS_2/WSe_2 Heterobilayer. Nat. Commun. 2023, 14, 4679. https://doi.org/10.1038/s41467-023-40329-3.
9. Jin, C.; Regan, E. C.; Wang, D.; et al. Identification of Spin, Valley and Moiré Quasi-Angular Momentum of Interlayer Excitons. Nat. Phys. 2019, 15, 1140–1144. https://doi.org/10.1038/s41567-019-0631-4.
10. Rossi, A.; Zipfel, J.; Maity, I.; et al. Anomalous Interlayer Exciton Diffusion in WS_2/WSe_2 Moiré Heterostructure. ACS Nano 2024, 18, 18202–18210. https://doi.org/10.1021/acsnano.4c00015.
11. Cho, B. W.; Ivanova, T. V.; Li, Z.; et al. Unveiling the Spin-Valley Structure of Dipolar Exciton Ladders in R-Stacked WSe_2/WS_2 Moiré Heterobilayers. arXiv 2026, arXiv:2607.07590. https://doi.org/10.48550/arXiv.2607.07590.

12. Michail, A.; Anestopoulos, D.; Delikoukos, N.; et al. Tuning the Photoluminescence and Raman Response of Single-Layer WS_2 Crystals Using Biaxial Strain. J. Phys. Chem. C 2023, 127, 3506–3515. https://doi.org/10.1021/acs.jpcc.2c06933.
13. Waheed, Y.; Shit, S.; Surendran, J. T.; et al. Large Trion Binding Energy in Monolayer WS_2 via Strain-Enhanced Electron-Phonon Coupling. Commun. Mater. 2025, 6, 86. https://doi.org/10.1038/s43246-025-00809-z.
